# Cell surface fluctuations regulate early embryonic lineage sorting

**DOI:** 10.1016/j.cell.2022.01.022

**Published:** 2022-03-03

**Authors:** Ayaka Yanagida, Elena Corujo-Simon, Christopher K. Revell, Preeti Sahu, Giuliano G. Stirparo, Irene M. Aspalter, Alex K. Winkel, Ruby Peters, Henry De Belly, Davide A.D. Cassani, Sarra Achouri, Raphael Blumenfeld, Kristian Franze, Edouard Hannezo, Ewa K. Paluch, Jennifer Nichols, Kevin J. Chalut

**Affiliations:** 1 Wellcome-MRC Cambridge Stem Cell Institute, Jeffrey Cheah Biomedical Centre, University of Cambridge, Puddicombe Way, Cambridge CB2 0AW, UK; 2 Centre for Trophoblast Research, University of Cambridge, Downing Street, Cambridge CB2 3EG, UK; 3 Living Systems Institute, University of Exeter, Exeter EX4 4QD, UK; 4 Cavendish Laboratory, Department of Physics, University of Cambridge, JJ Thomson Ave, Cambridge CB3 0HE, UK; 5 Institute of Science and Technology Austria, Am Campus 1, Klosterneuburg 3400, Austria; 6 MRC Laboratory for Molecular Cell Biology, University College London, London WC1E 6BT, UK; 7 Department of Physiology, Development and Neuroscience, University of Cambridge, Downing Street, Cambridge CB2 3DY, UK; 8 Gonville & Caius College, University of Cambridge, Trinity St., Cambridge CB2 1TA, UK

**Keywords:** cell sorting, lineage segregation, cell surface mechanics, cell surface dynamics, embryo development, morphogenesis

## Abstract

In development, lineage segregation is coordinated in time and space. An important example is the mammalian inner cell mass, in which the primitive endoderm (PrE, founder of the yolk sac) physically segregates from the epiblast (EPI, founder of the fetus). While the molecular requirements have been well studied, the physical mechanisms determining spatial segregation between EPI and PrE remain elusive. Here, we investigate the mechanical basis of EPI and PrE sorting. We find that rather than the differences in static cell surface mechanical parameters as in classical sorting models, it is the differences in surface fluctuations that robustly ensure physical lineage sorting. These differential surface fluctuations systematically correlate with differential cellular fluidity, which we propose together constitute a non-equilibrium sorting mechanism for EPI and PrE lineages. By combining experiments and modeling, we identify cell surface dynamics as a key factor orchestrating the correct spatial segregation of the founder embryonic lineages.

## Highlights

• EPI and PrE mechanically segregate in early mouse embryos
• Segregation is not explained by classical, static mechanical models
• Segregation is instead explained by a dynamic parameter, cell surface fluctuations
• Fluctuations link to cell fluidity, together comprising a non-equilibrium sorting model

## Graphical abstract

**Figure:**
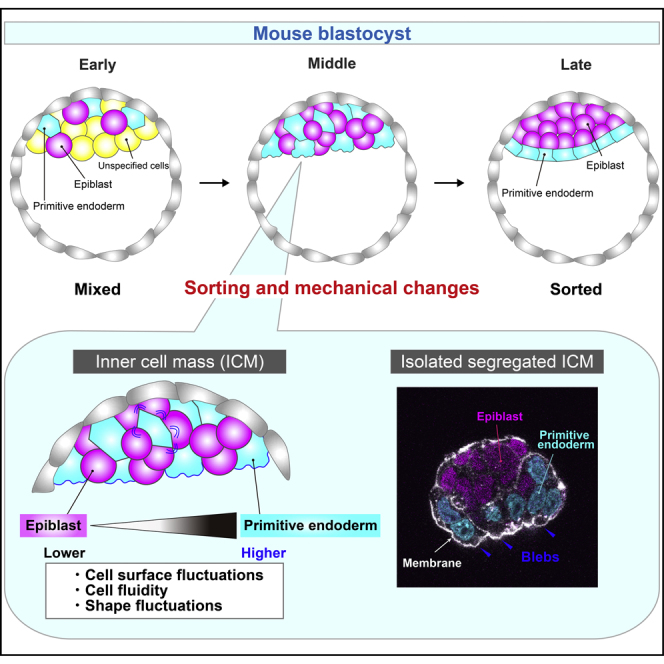

## Teaser

In early mammalian embryos differential cell surface fluctuations, rather than static physical parameters, account for the sorting of the primitive endoderm from the epiblast in the inner cell mass.

## Introduction

**Figure 1:**
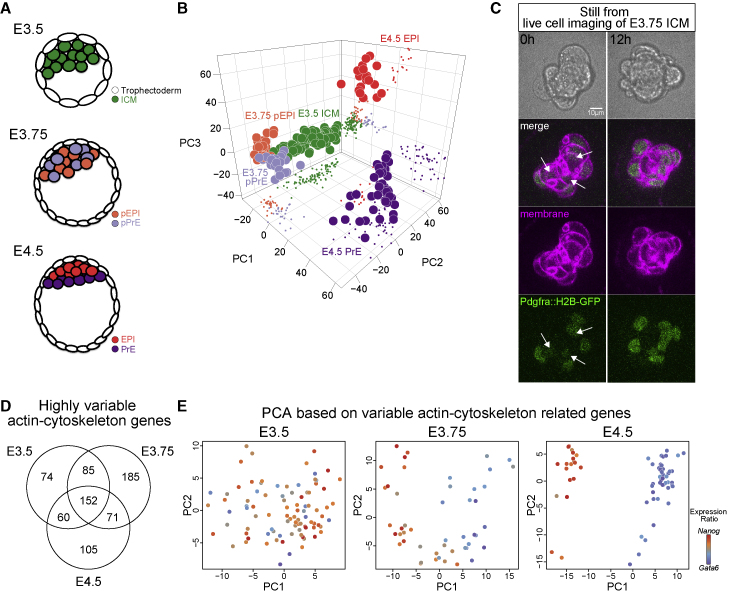
EPI and PrE begin to segregate at E3.75 (A) Schematic of EPI and PrE segregation in blastocysts. (B) Principal component analysis (PCA) plot of E3.5, E3.75, and E4.5 cells colored according to their stage. Each dot represents a single cell. (C) Images of an isolated E3.75 *Pdgfra*^*H2B-GFP/+*^*mTmG*^*+/−*^ ICM cultured *ex vivo*, taken as stills from movies (Video S1). T = 0 and 12 h show the EPI and PrE sorting stage and completed sorting stage, respectively. *Pdgfra*^*H2B-GFP*^ was expressed in PrE nuclei (green) and mTmG at the cell membrane (magenta). Arrows indicate that pPrE cells are located inside the ICM. See also Video S1. (D) Venn diagram of the number of highly variable actin-cytoskeletal genes in E3.5, E3.75, and E4.5 ICM cells. (E) PCA plot of E3.5, E3.75, and E4.5 ICM cells based on the highly variable cytoskeletal genes (E3.5: n = 371, E3.75: n = 493, E4.5: n = 388, log_2_ FPKM > 1, logCV^2^ > 0.5, see Table S1) colored according to the ratio of *Nanog* to *Gata6* expression. Each dot represents a single cell from ICM. See also Figure S1.

An essential event in the development of a mammal is the segregation of the epiblast (EPI), which will form the fetus, from the extraembryonic tissues that manage implantation, nutrition, and patterning of the fetus. The first step of this process is the formation of the blastocyst, which has been well described in mouse (Nishioka et al., 2009, 2008; Tarkowski and Wróblewska, 1967). The blastocyst forms as the outside cells of the pre-implantation embryo differentiate into trophectoderm (the source of the placenta) and cavitation occurs (Figure 1A). At this point, the inside cells comprising the inner cell mass (ICM) are aggregated and firmly adhered to the trophectoderm on the proximal pole of the blastocyst (Figure 1A, E3.5). Subsequently, a subpopulation of ICM cells becomes sensitive to fibroblast growth factor 4 (FGF4), heralding primitive endoderm (PrE) bias (Guo et al., 2010; Ohnishi et al., 2014). Within the uterus, and *ex vivo*, precursors of the EPI and PrE emerge in a spatially random manner (Chazaud et al., 2006; Plusa et al., 2008; Rossant et al., 2003). Coincident with identity acquisition, the cells physically sort, resulting in PrE establishing a single layer of cells covering the cavity-facing surface of the ICM with the EPI enclosed between the PrE and polar trophectoderm (Plusa et al., 2008). The chemical signaling requirements for fate specification are well understood: FGF4-dependent ERK activation is necessary and sufficient for PrE specification in the mouse (Yamanaka et al., 2010). Much less is known about how proper positioning of PrE is achieved. It is known that once all PrE cells are on the cavity-facing surface of the ICM, they polarize (Bassalert et al., 2018) and undergo aPKC-dependent epithelization (Saiz et al., 2013), but the mechanical means by which PrE cells segregate from the EPI in the first place and then remain on the cavity side until the PrE epithelializes (Figure 1A) remains a mystery. This work seeks to investigate the mechanical mechanisms by which the PrE sorts and remains segregated in the ICM.

Several general mechanical mechanisms for cell sorting have been proposed previously, including differential adhesion (Foty and Steinberg, 2004), differential surface tension (Lecuit and Lenne, 2007), and differential cell-cell affinity (Amack and Manning, 2012; Chan et al., 2017; Maître et al., 2012, 2016). Here, we examined all these possibilities in the context of sorting in the ICM and found that none of these mechanical mechanisms appeared sufficient to explain robust segregation of the PrE lineage from the EPI. Instead, through a combination of experiments and physical modeling, we uncovered that enhanced surface fluctuations in the PrE lineage are a key, intrinsically dynamic, mechanical factor in facilitating the segregation of these early embryonic lineages.

## Results

### EPI and PrE begin to segregate at E3.75

**Figure S1:**
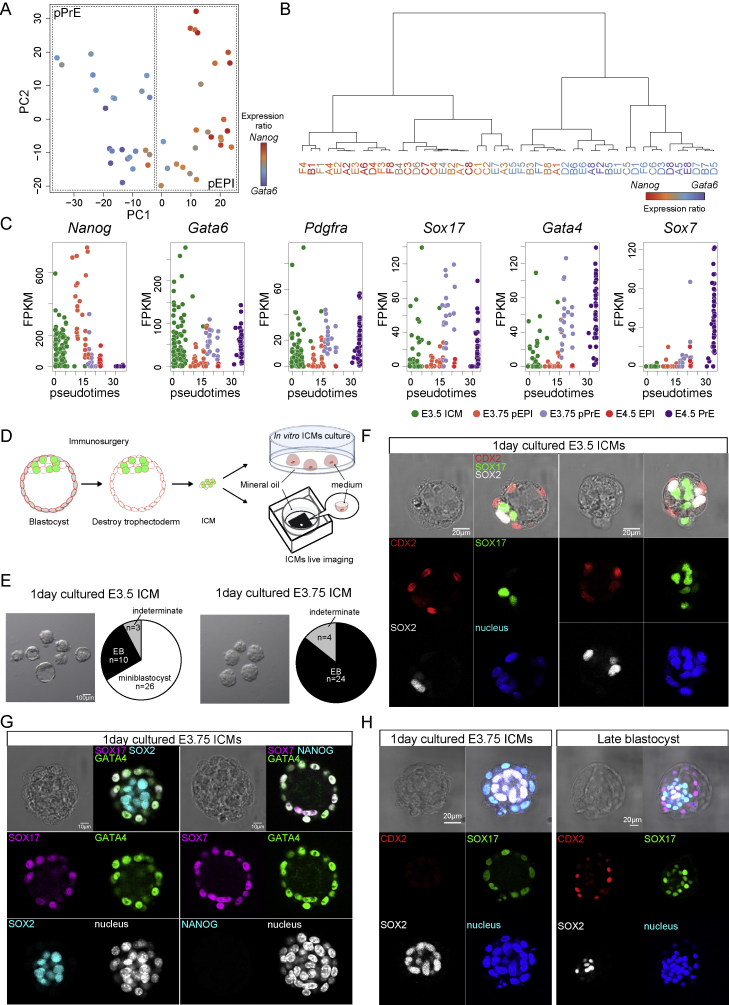
pEPI and pPrE begin to segregate at approximately E3.75, related to Figure 1 (A) PCA of E3.75 single cells computed with highly variable genes (n = 3259, log_2_ FPKM> 0.5, logCV^2^> 0.25) (B) Dendrogram of E3.75 ICM cells based on variable genes for E3.75 stage; expression coloured according to the ratio of *Nanog* to *Gata6* expression. (C) Expression profiles of selected genes ordered by pseudo time scale. (D) Schematic images of ICM isolation from blastocyst and culture. Sequential images of isolated E3.75 mTmG^+/-^*Pdgfra*^*H2B-GFP/+*^ ICM culture was performed in an embryo immobilisation chip using spinning disk confocal microscopy. (E) Bright-field images and the proportion of miniblastocysts, i.e. ICMs with a cavity embryoid bodies (EBs) and indeterminate morphologies of isolated E3.5 and E3.75 ICMs cultured for one day. Note that E3.75 cultured ICMs lose the capacity to form a mini-blastocyst. (F) Representative images of one day cultured isolated E3.5 ICMs. EPI marker, SOX2, PrE maker, SOX17 and TE marker, CDX2 were expressed in one day cultured E3.5 ICMs. (G) Immunofluorescence staining of isolated E3.75 ICMs cultured for one day from *Pdgfra*^*H2B-GFP/+*^. SOX2 is an EPI marker, and NANOG is an early EPI marker that is barely expressed in implantation stage embryos. SOX17 and GATA4 are PrE markers. SOX7 is late PrE marker. (H) Representative images of one day cultured isolated E3.75 ICMs and late-stage blastocyst. EPI marker, SOX2 and PrE maker, SOX17 were expressed in one day cultured E3.75 ICMs. TE marker, CDX2 was expressed in late-stage blastocyst but not in one day cultured E3.75 ICMs, indicating that, unlike E3.5 cultured ICMs, E3.75 cultured ICMs lose the capacity to make TE.

To determine the most relevant stage to investigate ICM sorting, we used RNA sequencing to analyze the gene expression of single ICM cells at E3.75 and combined it with previous analyses performed at E3.5 and E4.5 (Mohammed et al., 2017). Principal component analysis (PCA) revealed stage-specific clusters, indicating that in the E3.75 ICM, progenitors with specific embryo lineages, pEPI and pPrE, are just beginning to become distinct (Figures 1B and S1A–S1C).

To study the dynamics of segregation of pEPI and pPrE cells in E3.75 ICMs, we generated time-lapse movies of isolated ICMs from embryos expressing both a PrE lineage reporter, *Pdgfra*^*H2B-GFP*^ (Hamilton et al., 2003), and a plasma membrane-localized reporter, mTmG (Muzumdar et al., 2007) (Figure 1C; Video S1). With this analysis, we observed that pPrE cells that were initially randomly distributed sorted to the surface of the ICM.

Video S1. EPI and PrE sorting in isolated E3.75 ICM, related to Figure 1 Time-lapse sequence of isolated E3.75 mTmG+/-PdgfraH2B-GFP/+ 3 ICM in an embryo immobilization chip.

To identify a simplified system to study this lineage segregation, we first confirmed, as shown in Wigger et al. (2017), that pEPI and pPrE cells in ICMs isolated from E3.5 or E3.75 blastocysts can segregate and commit to EPI and PrE in culture without trophectoderm (Figure S1D). The majority of E3.5 ICM formed “miniblastocysts” (Figure S1E) containing cavities, with some external cells expressing the trophectoderm marker, CDX2 (Figure S1F). The later E3.75 ICMs formed embryoid body-like structures with no cavity or CDX2 expressing cells. After 1 day in culture, the PrE enveloped the EPI, confirmed using immunofluorescence (Figures S1G and S1H). Taken together, our data confirm proper fate segregation and maturation in isolated E3.75 ICMs in the absence of trophectoderm, and we conclude that E3.75 is the appropriate stage of the ICM to study what mechanical mechanisms drive sorting of the EPI and PrE.

### Differences in cell-cell adhesion, migration, and surface tension do not adequately explain cell sorting

**Figure S2:**
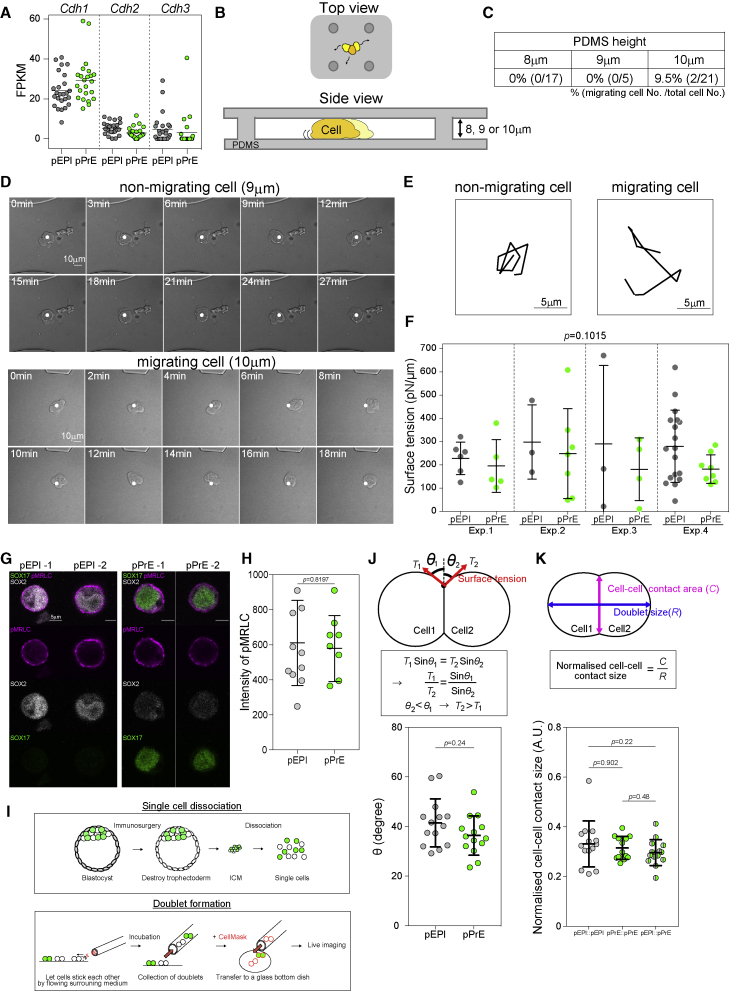
Differences in cell-cell adhesion, migration, and surface tension do not adequately explain cell sorting, related to Figure 2 (A) The mRNA expression level of *cdh1* (E-cadherin), *cdh2* (N-cadherin) and *cdh3* (P-cadherin) in E.3.75 pEPI and pPrE, indicating very little differential expression of adhesion factors between pPrE and pEPI. (B) Schematic of a polydimethylsiloxane (PDMS) confinement device for testing cell migration potential in confinement. (C) The proportion and number of E3.75 ICM cells migrating under 8, 9 or 10 μm height confinements, indicating that these cells have very little capacity to undergo confined migration. (D) Representative images of time series of non-migrating and migration E3.75 ICM cells using 9 μm or 10 μm height confinement. (E) Trajectories of the two E3.75 ICM cells in (D) during ten sequential frames. Note that 12 μm channels were also attempted but did not confine the cells. (F) The surface tension of dissociated pEPI and pPrE from E3.75 *Pdgfra^H2B-GFP/+^* embryos measured using an atomic force microscope (AFM), using the technique presented in (Chugh et al., 2017). P-value was calculated by 2-way ANOVA using cell type and experimental replicate as variables. (G) Representative images of pMRLC (magenta), a proxy for cytoskeletal tension, in E3.75 pEPI and pPrE, further indicating that there is little difference in surface tension between pEPI and pPrE. SOX2 is an EPI marker. SOX17 is a PrE marker. (H) The intensity of pMRLC in E3.75 pEPI and pPrE, quantified from the segmeneted signal across several isolated cells. (I) Schematic of the formation of pEPI and pPrE doublets. (J) Schematic of heterotypic doublet showing how external contact angle is measured, along with force balance equations indicating that the smaller angle of the two should possess a higher surface tension. The external contact angles of E3.75 pEPI and pPrE heterotypic doublets. (K) Schematic of doublet showing how normalised cell-cell contact size is measured. Normalised cell-cell contact size of E3.75 homotypic doublets (pEPI::pEPI, pEPI::pPrE) and heterotypic doublets (pPrE::pPrE). Error bars throughout figure correspond to the standard deviation of the data.

To understand what drives ICM sorting, we first considered two mechanisms suggested in the literature: differences in cell-cell adhesion or in migration (Bassalert et al., 2018; Chazaud et al., 2006). Importantly, cell-cell adhesion forces are generally known to play only a small role in tissue sorting (Amack and Manning, 2012; Maître and Heisenberg, 2011). Moreover, E-cadherin, an important regulator of cell-cell adhesion in early development (Halbleib and Nelson, 2006), has been shown not to be differentially distributed at the protein level and to be unnecessary for cell sorting in the blastocyst (Filimonow et al., 2019). We also found that E-cadherin is not differentially expressed between pEPI and pPrE at E3.75 (Figure S2A, showing that N- and P-cadherin (Cdh2 and Cdh3) are also not differentially expressed). Taken together, we conclude that differential cell-cell adhesion is unlikely to play more than a minor role in the sorting of the mouse ICM.

A contribution of directed migration to sorting is more difficult to rule out definitively. However, in images of ICM sorting (Plusa et al., 2008), there are no indications that pPrE cells in the ICM display protrusions suggestive of mesenchymal migration. Nevertheless, it is possible that pPrE cells in the ICM are capable of amoeboid migration, a migration mode often displayed by cells in confinement (Paluch et al., 2016), which could be difficult to detect from shape analysis alone. We thus assessed, using 3D confinement assays that facilitate migration of cells capable of amoeboid motility (Aspalter et al., 2020), the ability of ICM cells to migrate. We found that there was almost no detectable migration of E3.75 ICM cells, regardless of the level of confinement (Figures S2B–S2E), even though other cells types can migrate efficiently in similar conditions (Liu et al., 2015). This suggests that ICM cells do not have a high level of migration competence, making migration an unlikely candidate to drive robust cell sorting.

Given that lineage-specific differences in cell-cell adhesion and migration do not seem to be good candidates to propel robust cell sorting in the blastocyst, we turned to another candidate that has been suggested to drive cell sorting, cell surface mechanics (Meilhac et al., 2009). First, we probed our transcriptomics data for changes in the actin cytoskeleton and its regulators. E3.75 ICM cells, compared with ICM cells at other blastocyst stages, showed the most highly modulated actin-cytoskeleton-related genes (Figure 1D; Table S1). PCA of each stage based on variable actin-cytoskeleton-related genes showed that their expression became distinct at E3.75 (Figure 1E), coinciding with pEPI and pPrE cells sorting, suggesting that there may indeed be mechanical asymmetries arising between pPrE and pEPI at E3.75.

One possible manifestation of mechanical asymmetries, which has been previously proposed to mediate cell sorting, is differential cell surface tension (Lecuit and Lenne, 2007). To investigate cellular surface tension in the ICM, we first used an atomic force microscope (Chugh et al., 2017). The surface tension of pEPI and pPrE isolated from E3.75 ICMs was highly variable, but no significant differences were detected (Figure S2F). Cell surface tension predominantly reflects the contractile tension of the actomyosin cortex (Salbreux et al., 2012), which is primarily controlled by myosin II activity (Chugh and Paluch, 2018). We thus assessed the levels of phosphorylated myosin regulatory light chain (pMRLC), a key regulator of myosin activity (Heissler and Sellers, 2016). Upon measuring pMRLC levels, we found no difference between pEPI and pPrE (Figures S2G and S2H). These results strongly suggest that there is no significant difference in cortical tension between pEPI and pPrE cells. To further test this, we used a complementary approach to assess cortical tension differences, by analyzing the shape of heterotypic cell doublets of pEPI and pPrE (Figure S2I). In such doublets, the force-balance equation at the cell-cell contact (Figure S2J) implies that the cell with higher cortex tension displays a smaller contact angle. Yet, we found no statistically significant difference in contact angles displayed by pEPI and pPrE cells in heterotypic doublets (Figure S2K), and if anything, the pPrE doublets have smaller contact angles than pEPI doublets. Taken together with our AFM data and pMRLC images, we conclude that cortex tension differences are not likely a major factor in driving pEPI/pPrE sorting.

### Cell-cell affinity of pEPI is higher than pPrE, but cell-cell affinity differences alone are insufficient to lead to robust cell sorting

**Figure 2:**
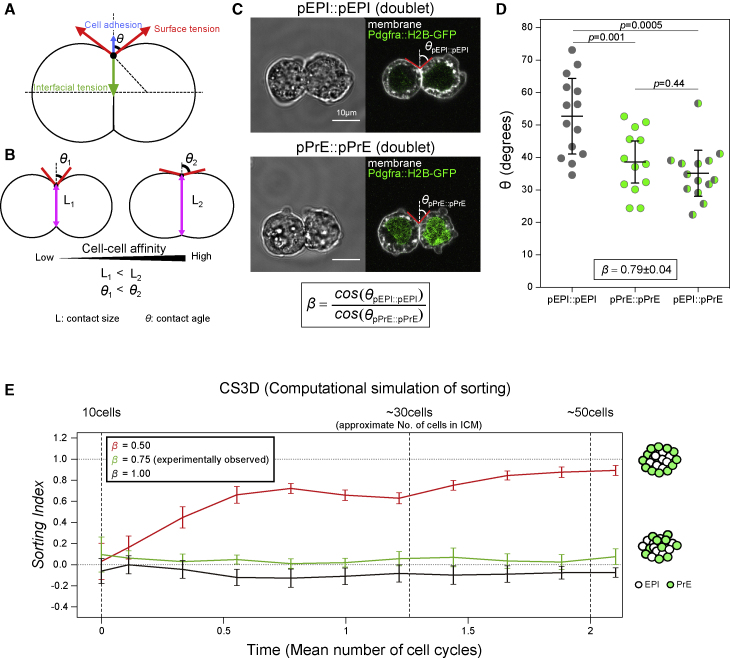
Cell-cell affinity of pEPI is higher than that of pPrE, but insufficient for cell sorting (A) Schematic of two ICM cells forming a doublet. The shape of the doublet is determined by the balance of surface tensions (dominated by cortical tension) at the cell-medium interface, interfacial tension at the cell-cell interface, and cell-cell adhesion forces. We note that cell-cell adhesion forces are small compared with the surface tension and can generally be neglected. (B) Schematic of cell-cell affinity in a cell doublet. (C) Representative images of a pEPI (pEPI::pEPI) and a pPrE (pPrE::pPrE) homotypic doublet, formed by dissociated single cells from E3.75 *Pdgfra*^*H2B-GFP/+*^ blastocysts. pPrE expressed *Pdgfra*^*H2B-GFP*^ at nuclei (green). Plasma membrane labeled with a membrane dye, CellMask Deep Red (false-colored white). pEPI or pPrE cells homotypic doublet showing how *β* is used as a measure for cell-cell affinity. *θ*_pEPI::pEPI_ is the pEPI::pEPI external contact angle, and *θ*_pPrE::pPrE_ is the pPrE::pPrE external contact angle. (D) *θ* of the different types of doublets that can be formed from E3.75 pEPI and pPrE cells. Each dot represents the mean of both sides of external contact angles. The data are combined over N = 3 independent experiments, and *β* was calculated from the mean *θ*_pEPI::pEPI_ and *θ*_pPrE::pPrE_ as 0.79 ± 0.04 from the equation in (C). p values calculated by two-way ANOVA using cell type and replicate number (N = 3) as variables. Error bars, here as in all figures, represent standard deviation. (E) 3D force-based cell-sorting simulation (CS3D) of EPI and PrE sorting applied with differential affinity ratio *β*. *β* = 1.0 indicating no difference in affinity between pEPI and pPrE. Our simulation assumes a system evolving from 10 to 50 cells, which represents slightly more than two cell divisions. Sorting index = 0.0 indicates random sorting, and Sorting index = 1.0 indicates complete sorting with PrE located on the outside. See also Figure S2.

Another suggested mechanical regulator of segregation in developing tissues is differential cell-cell affinity, which is determined by the force balance between cell-cell adhesion, cell surface tensions, and interfacial tension at cell contacts (Amack and Manning, 2012; Chan et al., 2017; Maître et al., 2012, 2016) (Figures 2A and 2B). Physical modeling suggests that in multicellular systems, differences in cell-cell affinity between two cell types can be sufficient to drive cell sorting (Revell et al., 2019). To analyze differential cell-cell affinity, two quantities describing the contact between two cells in a homotypic doublet of each cell type could be measured. The two quantities are differences in contact size (for simplicity we used contact length in max projection images) and differences in contact angle. Contact size is the most straightforward measure; however, for quantifying individual contributions to cell-cell affinity contact angles are useful since they can be used to compute forces more directly from the force-balance equations as they do not require additional geometrical assumptions (Figures 2B and S2J). Ultimately, both contact area and contact angles should be highly correlated and are equally good estimates for cell-cell affinity, and both are used in this work. To test whether differential cell-cell affinity could control ICM sorting, we first measured the external contact angles between pEPI and pPrE doublets made by aggregating two E3.75 *Pdgfra*^*H2B-GFP*^ ICM cells (Figures 2B and 2C). The external contact angles of homotypic pEPI cell doublets (pEPI::pEPI) were larger than those of both pPrE::pPrE and pEPI::pPrE (Figure 2D). An affinity parameter, *β*, between two cell types can be calculated based on these angles (Figure 2B; STAR Methods). The affinity parameter reflected the cell-cell affinity differential between different types of doublets and was found to be

(Equation 1) $$\beta =\frac{\cos\left(\theta_{\text{pEPI}∷\text{pEPI}}\right)}{\cos\left(\theta_{\text{pPrE}∷\text{pPrE}}\right)}=0.79\pm 0.04.$$

where the reported error is standard deviation. *β* < 1 indicates that the affinity is greater in pEPI::pEPI than pPrE::pPrE, suggesting that pPrE would be biased to sort to the outside of an aggregate. On the other hand, we also measured contact size directly and found very little difference between the pEPI::pEPI doublets and pPrE::pPrE homotypic doublets (Figure S2K), so it is possible that *β* is closer to 1 than our estimate. Nevertheless, to push the analysis forward, we assume the *β* we measured from our contact angle measurements, recognizing this is likely a lower extreme of *β* in this system.

To test whether the measured cell-cell affinity parameter is sufficient to account for the segregation of pEPI and pPrE cells, we used a 3D computational model based on the subcellular element method, termed 3D force-based cell-sorting simulation (CS3D, described in Revell et al. (2019) and also in STAR Methods). Briefly, each individual cell is modeled as a group of infinitesimal elements, interacting via nearest-neighbor forces. CS3D also incorporates cell growth and division and enables a multiscale modeling of inter- and intracellular interactions in multicellular aggregates such as the ICM. To score sorting in the aggregates, we established a sorting index (Revell et al., 2019; STAR Methods). The sorting index has a range of −1 to 1, where −1 indicates pEPI cells on the outside, 0 indicates random cell positioning (no sorting), and 1 indicates pPrE cells on the outside. With CS3D, contact size is a more straightforward measure of cell-cell affinity than contact angles, and we showed in previous work that contact size differences between two cell types is a very good predictor of whether or not those two cell types will sort in an aggregate comprising those cell types (see Figure 4 in Revell et al., 2019). Using CS3D, we simulated sorting in the ICM with the lowest extreme (i.e., highest affinity asymmetry, *β* = 0.75) of our experimentally observed value of *β* = 0.79 ± 0.04, with cells dividing from ∼10 to up to ∼50 cells. These numbers represent the approximate beginning and end number of cells in the ICM between E3.5 and E4.5, while the average ICM at mid-blastocyst stage possesses ∼30 cells (Artus et al., 2013). Surprisingly, even the most conservative estimate of differential cell-cell affinity we measured was insufficient to lead to pEPI and pPrE sorting in our simulations. A much stronger differential affinity (*β* < ∼0.5) was required in our simulations to efficiently sort cells within the experimental time frame (Figure 2E). Our model thus implies that the experimentally measured difference in static mean cell-cell affinity is insufficient to support robust sorting of pEPI and pPrE. Taken together with all the other results, this suggested we were missing a key parameter.

### pPrE displays higher surface fluctuations than pEPI

**Figure 3:**
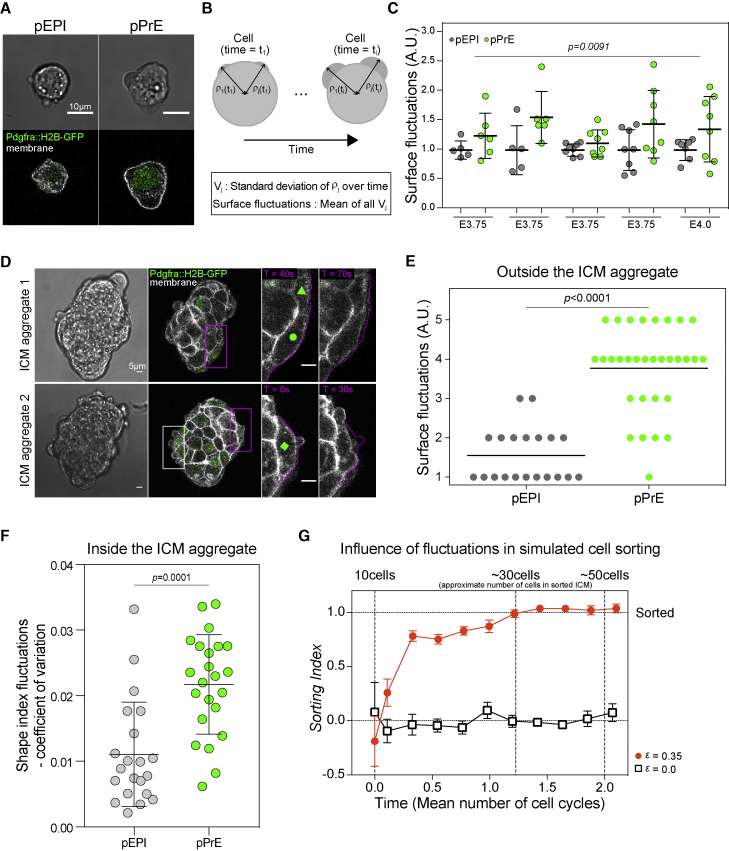
pPrE has higher surface fluctuation than pEPI (A) Representative images of dissociated E3.75 pEPI and pPrE cells from mTmG^*+/−*^*Pdgfra*^*H2B-GFP/+*^ mice, showing that pPrE, expressing expressed *Pdgfra*^*H2B-GFP*^ at nuclei (green), exhibits more blebbing than pEPI. Plasma membrane is labeled with membrane dye, CellMask deep red (white). (B) Schematic showing how surface fluctuations are calculated. (C) Single E3.75 and E4.0 pEPI and pPrE surface fluctuations. The amplitude of surface fluctuations was calculated using images every 10 s over a total of 5 min and normalized by the mean of pEPI for each time point and condition. p value calculated by two-way ANOVA using cell type and replicate number as variables, p value reported is for cell type (PrE versus EPI lineage). (D) Representative images of isolated ICM aggregations, each aggregation comprising 3 ICMs, from E3.75 mTmG^*+/−*^*Pdgfra*^*H2B-GFP/+*^ embryos, taken as stills from movies (see Video S6). Each outside cell was ranked single-blind from 1 to 5 by looking at the cell over the entire movie, 5 indicating a high level of surface fluctuations over the course of the movie and 1 indicating no visible surface fluctuations. Cells with green triangle, circle, and square indicating surface fluctuations = 2, 3, and 5, respectively. Purples boxes show enlarged regions in two rightmost panels. The white box outlines typical pPrE cells demonstrating their high degree of fluctuations in aggregates. (E) Blind rank analysis of surface fluctuations of pPrE and pEPI cells. (F) The coefficient of variation of shape index, representing fluctuations in cell shape, of E3.75 pEPI and pPrE cells from the inside of aggregated ICMs. p value calculated by one-way ANOVA. (G) Time series plot of CS3D simulation from 10 to 50 cells, assuming symmetric division, using the experimentally measured value of *β =* 0.75. *ε* = 0 means no difference in surface fluctuations between pEPI and pPrE. *ε* = 0.35 is the measured ratio of surface fluctuations of pPrE to pEPI. Each parameter set is averaged over N = 4 runs. The horizontal dotted line (sorting index *=* 1.0) shows the threshold beyond which core-shell sorting is complete, with pPrE on the outside. See also Figures S3 and S4 and Videos S2, S3, S4, and S6.

**Figure S3:**
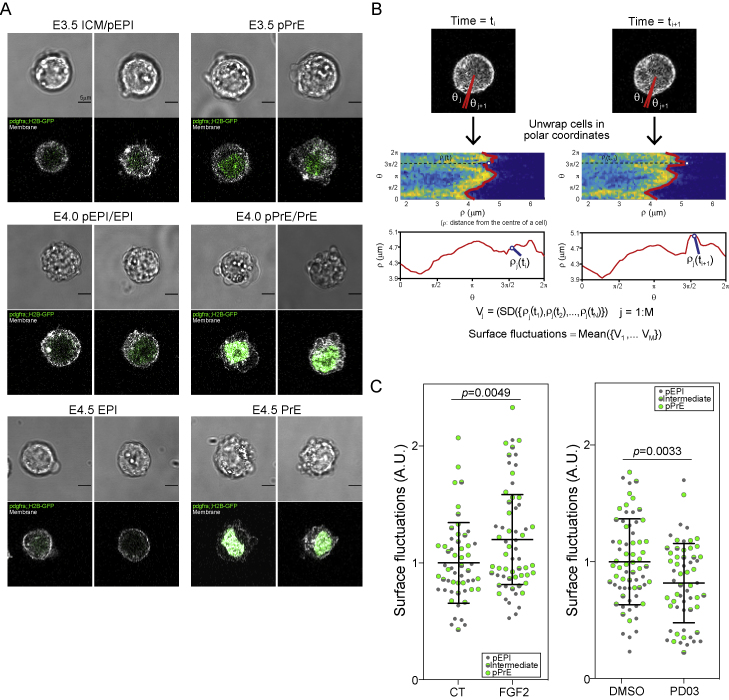
The PrE lineage blebs more than the pEPI lineage, related to Figure 3 (A) Representative images of isolated ICM cells from *Pdgfra^H2B-GFP/+^* E3.5, E4.0 and E4.5 blastocysts, indicating that at all stages the PrE lineage and its progenitors exhibit more blebbing than the EPI lineage and its progenitors. Note, however, that at E4.5, the blebs tend to present as smaller blebs, ruffles, or protrusions. Pdgfra starts to be expressed in pPrE at the early blastocyst stage. Pdgfra-negative E3.5 ICM cells are either pEPI or ICM cells, which have not specified their lineages yet. E4.0 ICM cells may contain pEPI, pPrE, EPI, and PrE. (B) Schematic outline of how surface fluctuation of a cell is measured from cell imaging data. (C) Single E3.75 pEPI and pPrE surface fluctuations with or without FGF2, 0.01% DMSO, or PD03 treated for < 45 minutes. Each plot is a combination of N = 3 independent experimental results. The amplitude of surface fluctuations was calculated using images every ten seconds over a total of five minutes. The amplitude was normalised by the total mean of CT or DMSO surface fluctuations in each individual experiments. P-value calculated by 3-way ANOVA using cell type, treatment, and replicate number as variables; reported p-value is for treatment.

Interestingly, consistent with early studies from the late blastocyst (Gardner and Papaioannou, 1975), we noticed that pPrE cells displayed a less smooth morphology compared with pEPI cells (Figures 3A and S3A). We thus asked whether these differences reflected increased dynamic cell surface fluctuations in pPrE, which could imply higher fluctuations in cell surface mechanics. To answer this, we isolated pEPI and pPrE cells from several stages of early ICM, including E3.75, and live imaged them for 5 min. We then quantified the amplitude of surface fluctuations displayed by the cells (Figures 3B and S3B; STAR Methods). Single pPrE cells exhibited significantly larger surface fluctuations than pEPI (Figure 3C). We then treated cells from the ICM with exogenous FGF, the primary instructive signal for PrE specification (Yamanaka et al., 2010; Figure S3C). Treatment with exogenous FGF also led to higher surface fluctuations in the ICM cells, and inhibition of ERK, the downstream effector of FGF signaling, reduced surface fluctuations in ICM cells (Figure S3C; Videos S2 and S3). Notably, our quantification does not distinguish between different types of cellular protrusions, but visual assessment revealed that the primary manifestation of the surface fluctuations was blebbing (Videos S4 and S5; Figure S3A).

Video S2. Surface fluctuations of isolated E3.75 pEPI with FGF2, related to Figure 3 Time-lapse sequence of isolated E3.75 mTmG+/-PdgfraH2B-GFP/+ 7 pEPI with FGF2 on PDL-coated dish. This file was assembled at the middle section of the cell every ten seconds over five minutes.

Video S3. Surface fluctuations of isolated E3.75 pPrE with FGF2, related to Figure 3 Time-lapse sequence of isolated E3.75 mTmG+/-PdgfraH2B-GFP/+ 11 pPrE with FGF2 on a PDL-coated dish. This file was assembled at the middle section of the cell every ten seconds over five minutes.

Video S4. Surface fluctuations of isolated E3.75 pEPI, related to Figure 3 Time-lapse sequence of isolated E3.75 mTmG+/-PdgfraH2B-GFP/+ 15 pEPI on PDL-coated dish. This file was assembled at the middle section of the cell every ten seconds over five minutes.

Video S5. Surface fluctuations of isolated E3.75 pPrE, related to Figure 3 Time-lapse sequence of isolated E3.75 mTmG+/-PdgfraH2B-GFP/+ 19 pPrE on a PDL-coated dish. This file was assembled at the middle section of the cell every ten seconds over five minutes

To examine whether differences in surface fluctuations were observable in the multicellular context, we monitored cellular membrane dynamics in ICMs isolated from blastocysts. Surface fluctuations were clearly visible on the outer edge of cell aggregates. In order to ensure a sufficient number of cells located on the outside layer to perform a quantification of surface fluctuations, we aggregated three isolated ICMs from E3.75 mTmG^*+/*−^*Pdgfra*^*H2B-GFP/+*^ blastocysts (Figure 3D). We then performed a blinded quantification of surface fluctuations in the ICM aggregates and found that pPrE cells on the outer layer of the ICM aggregate exhibited significantly higher surface fluctuations than similarly located pEPI cells (Figure 3E; Video S6). In the bulk of the ICM, no blebs or protrusions were visually evident along cell-cell contacts. However, contacts with neighboring cells would likely preclude protrusion extension within the dense ICM. If there are dynamics changes in cell surface mechanical properties, we reasoned this should be visible by studying changes in cell shape within the bulk. To assess possible shape fluctuations within the bulk, we used our movies of aggregated ICMs to first assess cell shape for both pEPI and pPrE over time. To assess cell shape, we measured the cell shape index (Bi et al., 2015; Park et al., 2015), which is a measure of cell elongation, defined in 2D as cell perimeter divided by the square root of the area (for example, the cell shape index of a circle is ∼3.54). We then used the coefficient of variation of the shape index over time as a proxy for shape fluctuations within a multicellular aggregate. We found that the coefficient of variation of the cell shape index was higher for pPrE than for pEPI, suggesting that cell surface fluctuations are also significantly higher for pPrE compared with pEPI in the bulk of the ICM (Figure 3F). Taken together, our data strongly suggest that the PrE lineage demonstrates significantly more dynamic cell surface mechanics than the EPI lineage, and they are likely a result of the intracellular signaling that initiates PrE lineage specification.

Video S6. Surface fluctuations of E3.75 ICM aggregates, related to Figure 3 Time-lapse sequence of isolated E3.75 mTmG+/-PdgfraH2B-GFP/+ 1 ICM aggregates. This file was assembled at one Z-section of the aggregation of every 20 seconds over five minutes. Left: ICM aggregate 1. Right: ICM aggregate 2.

**Figure S4:**
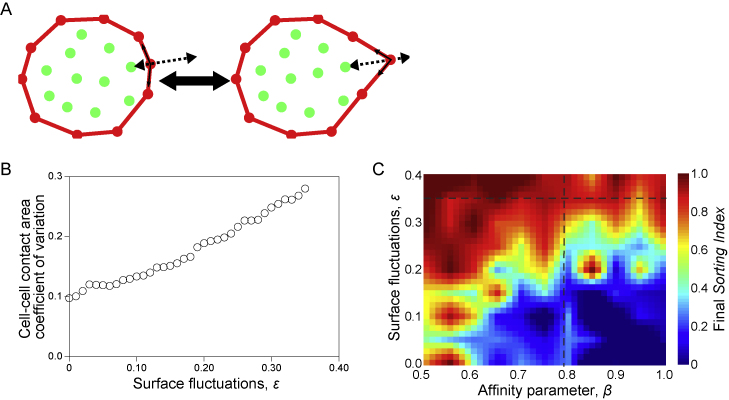
Surface fluctuations can be incorporated into the CS3D model, related to Figure 3 (A) Schematic of a cell in the CS3D method, showing elements as circles with colours indicating the part of the cell (red are surface elements and green are bulk, or cytoplasmic elements). Blebs are implemented by varying the surface tension in the simulated cell (consult methods for more details). (B) CS3D simulations showing the coefficient of variation of cell-cell contact area of a homotypic doublet (i.e. a doublet in which both simulated cells have the same surface tensions) as a function of the amplitude of surface fluctuations, *ε*. The CS3D simulations have a stochastic component so at this resolution of *ε* there is significant variability; thus, we used a rolling average with a window of 5 to visualise the data. (C) Phase space of the final sorting index for aggregates allowed to develop from 10 to 30 cells in *ε* and *β* space, with a resolution of 0.05 on each axis. The dotted lines (*ε* = 0.35, *β* = 0.79) represent the approximate experimentally measured parameters.

We then speculated that differences in surface fluctuations could contribute to segregating pEPI and pPrE cells. We assessed this hypothesis using physical modeling. To this aim, we extended the CS3D simulations to incorporate cell surface fluctuations by implementing random fluctuations in surface tension (Figure S4A). This fluctuation in surface tension simulates blebs in cells with a free surface and fluctuations in cell shape in the bulk of an aggregate (Figures S4A and S4B). We introduced a dimensionless parameter *ε* representing the ratio of surface fluctuations in pPrE to pEPI, and using the measured surface fluctuations of each cell type (from Figure 3C), we estimated *ε* ≈ 0.35. We then used CS3D to simulate pEPI and pPrE cell sorting with *β* = 0.75 and *ε* = 0, corresponding to equal surface fluctuations in PrE and EPI cells, or the experimentally observed fluctuations differential *ε* = 0.35. No sorting was observed for *ε* = 0. However, we observed thorough and robust sorting for *ε* = 0.35 (Figure 3G). We then assembled a phase space of the sorting index for the range *β* = [0.5, 1.00] and *ε* = [0.0, 0.40] to cover a wide range of experimental parameters. It is clear from the phase space that, though we see moderate segregation without a fluctuation differential as *β* approaches sufficiently extreme values of ∼0.5, the cells are capable of sorting even if *β* = 1 provided the pPrE cells have significantly larger surface fluctuations (Figure S4C). Thus, our model suggests that a differential in surface fluctuations could control the robust sorting of pEPI and pPrE.

### Variability in cell surface mechanics controls cell surface fluctuations

**Figure S5:**
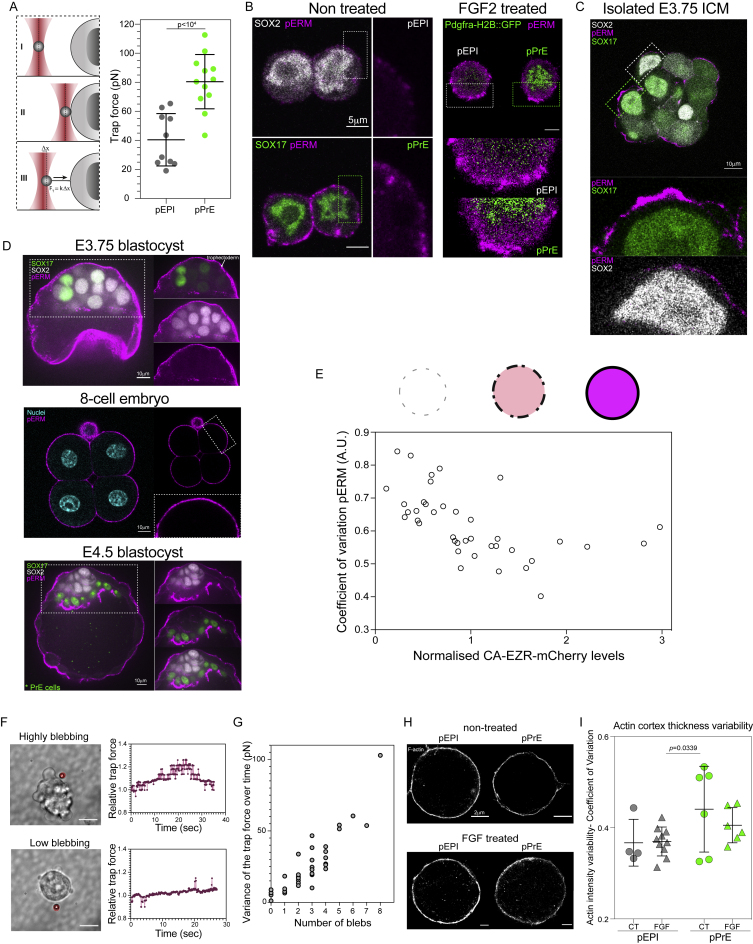
pERM and membrane tension variability is likely responsible for enhanced surface fluctuations in PrE lineage, related to Figure 4 (A) Schematic of optical tweezers to measure membrane tension in a cell. The membrane tension, as measured by trap force, of E3.75 pEPI and pPrE isolated from *Pdgfra^H2B-GFP/+^* embryos. P-value was calculated by 1-way ANOVA. (B) Representative images of pERM expression in pEPI and pPrE (magenta) with or without FGF2. pEPI expressed SOX2 (white). pPrE expressed SOX17 or *Pdgfra^H2B-GFP^* at nuclei (green). pERM is clearly more highly expressed, and highly variable at the surface, in pPrE. (C-D) Representative images of pERM in E3.75 isolated ICM, E3.75 blastocyst, 8-cell embryos and E4.5 blastocyst, indicating that pERM is much more variable on the surfaces of ICM cells at E3.75 than other stages and lineages in the early embryo. (E) Schematic showing how pERM expression changes depending on CA-EZR expression. The coefficient of variation of pERM intensity along cell the surface is plotted against the intensity of mCherry. (F) Left, representative images of membrane tension measurement of an ESC that is highly blebbing (top) and of a cell that is lowly blebbing (bottom) using optical tweezers. Right, plot displaying the relative trap force measured over time of the corresponding cells displayed on the left. Higher blebbing cells display higher variance of trap force over time compared to low blebbing cells. The scale bars represent 10 μm. A red target has been placed at the initial position of the bead before tether formation to help visualize bead displacement. (G) The variance of the trap force over time and the number of blebs in a cell, indicating a strong correlation between the variance of membrane tension and blebbing. (H) Representative stochastic optical reconstruction microscopy (STORM) images of actin in *Pdgfra^H2B-GFP/+^* E3.75 pEPI and pPrE with and without FGF2. (I) Coefficient of variation of F-actin thickness in E3.75 pEPI and pPrE with and without FGF2 representing how variable the actin cortex was. Each dot represents a single cell. P-value was calculated by 2-way ANOVA using cell type and treatment as variables. Notably, there was no significant difference in variability when treating with FGF; however, the thickness of the cortex was significantly greater when the cells were treated with FGF (191 nm compared to 155 nm, p << 10^-4^). We also note that the preparation for STORM requires many wash steps that may result in the cells with the most blebs being washed off.

We then asked which cellular mechanisms led to enhanced surface fluctuations in pPrE cells. As the fluctuations manifested mostly as blebs, the most likely candidate mechanisms are cortical tension, which generates intracellular pressure that drives bleb expansion, or effective membrane tension, which resists any cellular deformation and thus acts against bleb formation (Charras and Paluch, 2008). As the static mean cortical tension does not appear to be significantly different between EPI and PrE lineages (Figures S2G and S2H), overall differences in cortical tension are unlikely to play a prominent role. Thus, we turned to effective membrane tension, which depends on the in-plane tension of the lipid bilayer and membrane attachments to the underlying cortex (Pontes et al., 2017). We measured effective membrane tension using optical tweezers (De Belly et al., 2020), and found, surprisingly, that it is higher in pPrE than in pEPI cells (Figure S5A). Effective membrane tension is primarily regulated by the level of membrane-to-cortex attachment, with the Ezrin-Radixin-Moesin (ERM) protein family (Fehon et al., 2010) playing a key role. Correspondingly, we found that there were much higher overall levels of phosphorylated ERM (pERM), the active form of ERMs, in pPrE than pEPI cells (Figure S5B). These observations appeared counter-intuitive at first, as high membrane tension generally limits blebbing (Charras and Paluch, 2008). Significantly, however, the optical tweezer measurement is a highly local measurement, and along with overall higher pERM levels, we observed that there was also a high degree of spatial variability in pERM levels along the cell boundary in pPrE cells (Figure S5B). Furthermore, we observed high pERM spatial heterogeneity in the outer layer of the ICM of the E3.75 blastocyst (Figures S5B and S5C). This was especially noticeable when the pERM signal was compared with the 8-cell or E4.5 embryos, or the trophectoderm of the E3.75 blastocyst, in which the pERM signal was fairly continuous along the cell surfaces (Figure S5D).

**Figure 4:**
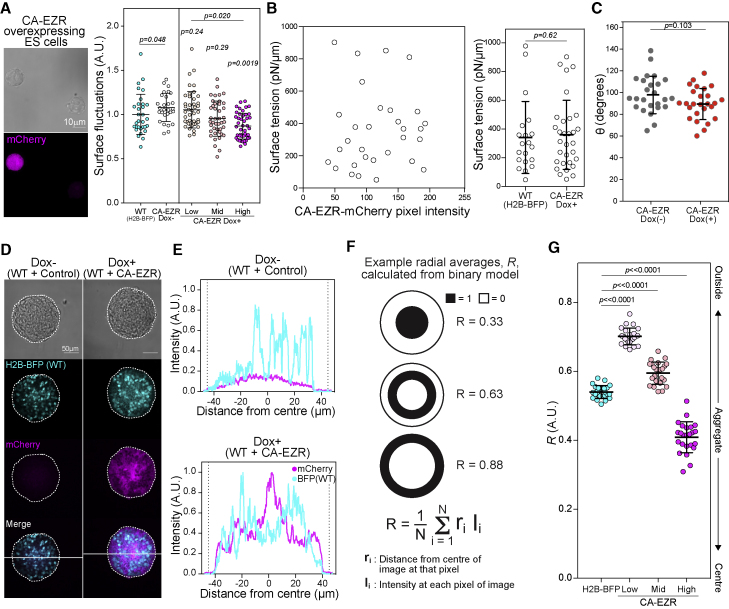
Differences in ezrin-mediated surface fluctuations regulate cell sorting (A) Representative images of constitutively active Ezrin-IRES-mCherry (CA-EZR) ES cells, showing a high degree of pERM variability in the low mCherry-expressing ES cells. Surface fluctuations of single CA-EZR cells without Dox and WT H2B-BFP, and CA-EZR ES cells with or without Dox in 2i+LIF. L, M, and H indicate low, medium, and high expression of mCherry as assessed by the 3-quantiles of expression in the mCherry-expressing cells. Surface fluctuations were normalized by the mean of the Dox− surface fluctuations in each of the experiments or the mean of the WT H2B-BFP surface fluctuations. p values were calculated using one-way ANOVA, with the p values above each group representing the outcome of pairwise comparison with Dox−, and the p value above all values in CA-EZR Dox+ condition representing the comparison of all groups. (B) The surface tension of dissociated Dox-treated CA-EZR ES cells measured using the AFM technique presented in Chugh et al. (2017) is plotted against the intensity of mCherry to show that there is no correlation between CA-EZR expression and surface tension. On the right is the surface tension of dissociated WT H2B-BFP ES cells and Dox-treated CA-EZR ES cells. p value was calculated by two-way ANOVA using cell type and experimental replicate as variables. (C) *θ* of the homotypic doublets that can be formed from CA-EZR ES cells with or without Dox. (D) Representative images of CA-EZR ES cells and WT H2B-BFP ES cells aggregated with or without Dox. The line drawn through the center of the aggregates represents the line over which we found an intensity profile in (E). (E) Representative comparison of BFP and mCherry line scan signals in the CA-EZR and H2B-BFP ES cells aggregates with or without Dox, using the line across the images in (D). (F) Schematic showing how the radial average (dipole moment) *R* is calculated, along with model examples of *R* for distributions shown. (G) *R* of aggregates of CA-EZR and H2B-BFP ES cells. See also Figure S5.

To further establish a link between pERM spatial heterogeneity and ERM activity levels, we used a doxycycline (Dox)-inducible constitutively active ezrin (EzrinT567D-IRES-mCherry, or CA-EZR for short) mouse ES cell line (De Belly et al., 2020). Using this line, we found that cellular mCherry levels anticorrelated with the variability of pERM in the membrane (Figure S5E), indicating that the higher the levels of CA-EZR, the less variable the pERM is at the membrane. We also found that upon Dox induction, mCherry expression anticorrelated to surface fluctuations across a broad range of mCherry values (Figure 4A), indicating that increasing the levels of active ezrin beyond endogenous levels in ES cells, and thus decreasing pERM heterogeneity, leads to a reduction in surface fluctuations.

We then sought further evidence that surface fluctuations reflect dynamics in cell surface mechanics. For this, we first measured the variability in effective membrane tension and also counted the number of observable blebs over time in several cells. We found that the temporal variability in membrane tension at the single-cell level highly correlates with the amount of blebbing in the cell (Figures S5F and S5G). Then, we used STORM microscopy to visualize the actin cortex in pEPI and pPrE and found a higher variation in the thickness of the actin cortex of pPrE cells versus pEPI cells (Figures S5H and S5I). Importantly, heterogeneities in ERM levels and in cortex organization can both promote blebbing (Charras and Paluch, 2008). Taking these observations together with the spatial heterogeneity of pERM in the PrE lineage, we speculate that differences in the variability, rather than in static mean values of cell surface mechanical parameters, account for the higher cell surface fluctuations observed in pPrE cells compared with pEPI cells.

### Cell surface fluctuations regulate sorting in ES cell aggregates

In order to experimentally test our hypothesis that surface fluctuations regulate sorting, we used our CA-EZR ES cell line to provide a cell system possessing a range of surface fluctuations (Figure 4A). We confirmed that the CA-EZR expression did not affect cortical tension or cell-cell affinity, which may have been confounding factors in our physical model (Figures 4B and 4C). Using the CA-EZR ES cells, we performed cell aggregation assays to directly assess how cell surface fluctuations affect sorting. As a control, we used an H2B-BFP ES cell line that displays slightly lower levels of cell surface fluctuations compared with the CA-EZR line (Figure 4A). We mixed the control ES cells with the CA-EZR ES cells at a 1:1 ratio and cultured these aggregates for 1 day with or without Dox (Figures 4D and 4E). We then quantified sorting by calculating the normalized average distance of the mCherry signal from the center of the aggregate, *R* (Figure 4F). Using this measure and thresholding to determine low-, mid-, and high-expressing CA-EZR cells, we found that low-expressing CA-EZR cells, which have enhanced surface fluctuations compared with controls, were preferentially found on the outside of the aggregate, while high-expressing CA-EZR cells, which have reduced surface fluctuations compared with controls, localized to the inside of the aggregate (Figures 4D and 4G).

**Figure 5:**
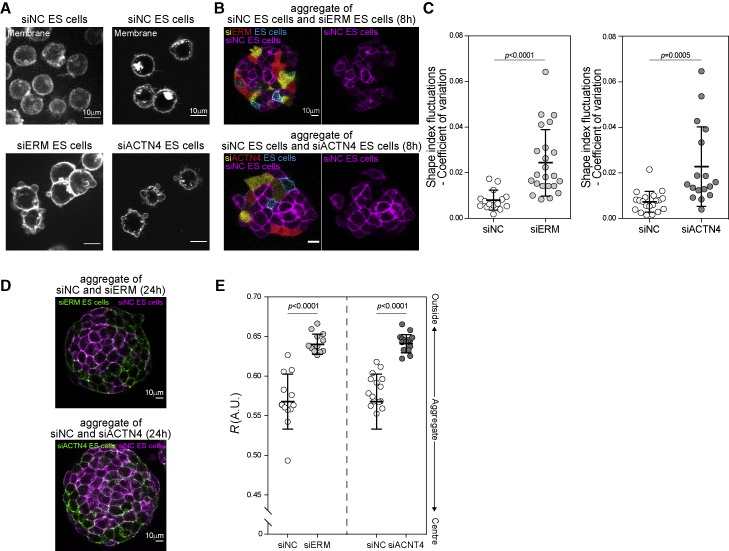
Cell surface mechanics perturbations lead to sorting in ES cell aggregates (A) Representative images of negative control siRNA-treated ES cells (siNC), ezrin, radixin, and moesin-siRNA-treated ES cells (siERM), and ACTN4-siRNA-treated ES cells (siACTN4). (B) Representative images of siNC ES cells (membrane labeled with mCherry) aggregated with either siERM or siACTN4 confetti ES cells. Images were acquired at 8 h after aggregation to ensure that the aggregates had not yet sorted. (C) The coefficient of variation of cell shape index of siNC, siERM, or siACTN4-treated ES cells. (D) Representative images of siNC ES cells (membrane labeled with mCherry) aggregated with either siERM or siACTN4 confetti ES cells. Images were acquired at 24 h after aggregation to ensure the aggregates had sufficient time to sort. (E) *R* of aggregates of siNC-treated ES cells aggregated with siERM or siACTN4 ES cells.

To further test the role of cell surface fluctuations in cell sorting, we developed two different perturbations to increase cell surface fluctuations. The first was siRNA-mediated triple knockdown of ERM (siERM), as depleting ERM decreases effective membrane tension (Fehon et al., 2010), leading to enhanced blebbing, while having little effect on cortical tension (Diz-Muñoz et al., 2010). The second was siRNA-mediated knockdown of α-actinin-4 (ACTN4) (siACTN4), an actin cross-linker. Indeed, we observed that both siERM and siACTN4 caused individual ES cells to display considerably more blebbing (Figure 5A). We then formed cell aggregates comprising either siACNT4 or siERM ES cells with negative control siRNA (siNC)-treated ES cells. In both cases, knockdown cells in aggregates displayed an increased coefficient of variation in cell shape index, suggesting that surface fluctuations were also enhanced in the bulk of aggregates (Figures 5B and 5C). Furthermore, in both aggregates containing siACTN4 and siERM, the knockdown cells with higher surface fluctuations sorted toward the outside of the aggregate (Figures 5D and 5E). Taken together, our data show that the position of cells within an aggregate is highly influenced by their level of surface fluctuations, thus strongly supporting our hypothesis that differences in surface fluctuations lead to cell sorting in multicellular aggregates.

### Cell surface fluctuations regulate sorting in the ICM

**Figure 6:**
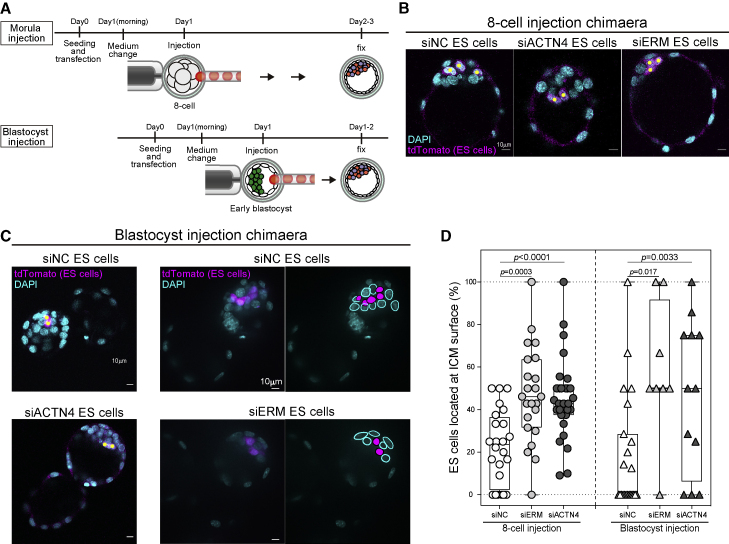
Cell surface fluctuations regulate sorting in the ICM (A) Schematic showing how 8-cell stage embryo and blastocyst injection of ES cells are conducted. (B) Representative images of 8-cell embryo injection of siNC, siACTN4, or siERM ES cells chimera at E4.0. Nuclei were stained with DAPI (cyan). ES cells were labeled with tdTomato (magenta). (C) Representative images of blastocyst injection of siNC, siACTN4, or siERM ES cells chimera at E4.0. Nuclei were stained with DAPI (cyan). ES cells were labeled with tdTomato (magenta). (D) The ratio of injected siNC, siACTN4, or siERM ES cells located at the surface of the ICM in the chimera blastocysts. For further details about the number of embryos, live cells, etc., see Table S2 and Video S7.

To test whether surface fluctuations also control sorting in the context of the ICM, we used an approach in which we injected ES cells and subsequently evaluated their chimeric contribution to the embryo. ES cells are a good model system, since they maintain pre-implantation EPI identity *in vitro* (Boroviak et al., 2014), and they are highly amenable to genetic manipulation at scale. Moreover, ES cells injected into the embryo anytime before the early mid-blastocyst stage (∼E3.5) should almost exclusively localize to the EPI on the inside of the ICM by E4.0 (Alexandrova et al., 2016; Posfai et al., 2021). We hypothesized that if we performed perturbations on ES cells to increase their cell surface fluctuations and then injected them into the ICM prior to E3.5, instead of localizing on the inside of the ICM as usual, they would localize toward the blastocoel side of the ICM, which we refer to as outside the ICM. To test this hypothesis, we utilized two different experimental paradigms probing chimeric incorporation of ES cells into the ICM. First, to test whether ES cells with higher surface fluctuations would sort from the inside to the outside of the ICM, we injected ES cells into the 8-cell stage embryos (E2.5) (Figures 6A and 6B). Next, to test whether ES cells with higher surface fluctuations would be incorporated into the inside of the ICM with the EPI or be sequestered to the outside of the ICM, we injected ES cells into the cavity of the early blastocyst (E3.25–E3.5) (Figures 6A and 6C). For both injection scenarios, we injected siNC, siERM, or siACTN4 treated ES cells and then assessed the cultured blastocyst at ∼E4.0 (Figures 6B and 6C), by which time injected control ES cells should populate the EPI (Alexandrova et al., 2016). Indeed, we found that for both 8-cell embryo and blastocyst injection, siNC cells were primarily situated in the sorted EPI. In contrast, we found that a significant fraction of both siERM and siACTN4 cells, in both injection scenarios, were integrated in the ICM, but in the outer layer corresponding to PrE localization adjacent to the blastocoel (Figures 6B–6D; Table S2). Importantly, the ES cells localized on the outside were expressing EPI marker Oct4 but not the early PrE lineage marker Sox17, so these perturbations are affecting positioning but not fate (Video S7). Thus, ES cells displaying enhanced surface fluctuations displayed disrupted sorting when injected into the mouse embryo, strongly supporting our hypothesis that surface fluctuations promote physical sorting of early embryonic lineages.

Video S7. 3D images of siRNA-treated ES cells chimeras, related to Figure 6 Whole z-stack images of (A) siNC-, (B) siERM- or (C) siACTN4- treated ES cells chimaera at E4.0. DAPI (blue), ES cells (red), SOX17 (green).

### Physical basis for the link between the mechanics of sorting and cell surface fluctuations

Our experiments and CS3D model suggest that cell sorting in the ICM does not follow a classical “thermodynamic” picture of phase segregation in which cells would segregate due to differences in static mean cell-cell affinity to lower the total free energy of the system. Instead, our data suggest that differences in cell surface dynamics, which manifest as differential cell surface fluctuations, play a key role. Nevertheless, it remains unclear how differential surface fluctuations might physically drive the sorting of cells. Notably, surface fluctuations are an intrinsically out-of-equilibrium process. Recently, another non-equilibrium process grounded not in classical parameters such as differential cell adhesion or cell-cell affinity, but instead on differences in cell shape, has been proposed to give rise to local demixing in multicellular aggregates. This proposal was based on vertex models, which simulate the dynamic behavior of cell aggregates (Farhadifar et al., 2007). A key input parameter in vertex models is the “preferred” perimeter each cell would strive to attain at equilibrium. It is not always possible for each cell to reach this preferred shape, which generates mechanical frustration and prevents the system from settling into a unique ordered equilibrium state. Interestingly, simulations of two cell populations with different preferred perimeters showed local demixing, driven by a non-equilibrium process (Sahu et al., 2020). Specifically, more “fluid” cells (which possess large perimeters, or shape indices) can squeeze and diffuse through the tissue much more easily than the less fluid cells. Moreover, the more fluid cells are kinetically disfavored to penetrate clusters of more “solid” cells (which possess smaller shape indices). The uneven energy barriers that exist between cells of different shape indices promote demixing of the two populations (Sahu et al., 2020). Interestingly, another modeling study has suggested that fluctuations could be related to tissue-level fluidity (Bi et al., 2016); we thus speculated there may be a link between cell fluidity and surface fluctuations.

**Figure 7:**
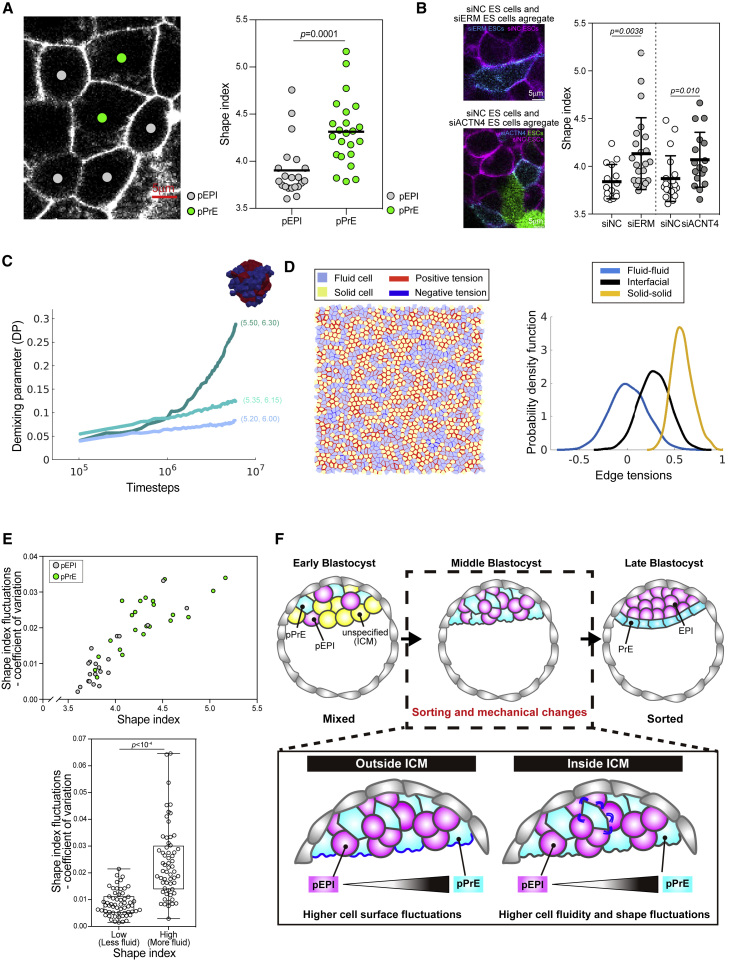
Physical modeling the link between the mechanics of sorting and cell surface fluctuations (A) Representative images of E3.75 pEPI and pPrE shape in isolated ICM aggregates cultured *ex vivo*, taken as stills from movies. Shape index of E3.75 pEPI and pPrE cells in an isolated ICM aggregates. (B) Representative images of aggregates of siNC ES cells (membrane labeled with mCherry) with either siERM or siACTN4 confetti ES cells. Shape index of aggregates of siNC with siERM or siACTN4 confetti ES cells. (C) Demixing parameter (DP) is quantified in mixtures of N = 216 cells in a fully three-dimensional confluent Voronoi model. Cell shape index for a polyhedra with surface area S and volume V is non-dimensionalized as S/Vˆ2/3 (Merkel and Manning, 2018). The mixture is composed of two subtypes with a fixed shape difference of 0.8, curves colored from blue to green in increasing order of average preferred shape index from (5.20, 6.00), (5.35, 6.15), and (5.50, 6.30), versus log(time). (D) Edge tensions in a confluent two-dimensional monolayer where the two cellular subtypes have a shape difference of 0.4. Yellow and blue cells have shape index 3.65 (solid-like) and 4.05 (fluid-like), respectively. The figure on top shows the line tension map—red for positive (contractile) and blue for negative (extensile) tension values. The system with N = 1,600 cells starts initially mixed and evolves to a ground state after FIRE minimization (as outlined in Bitzek et al., 2006). The histograms depict fluid-fluid, solid-solid, and solid-fluid interfacial edge tension values. (E) The coefficient of variation of shape index for E3.75 pEPI and pPrE cells, indicating the high degree of scaling with shape index. At bottom, the coefficient of variation of shape index for all E3.75 ICM cells and all ES cells, both untreated and treated, across all experiments. For all cells, the shape index was divided between low and high fluctuations by the median of the data (∼3.9). (F) Schematic indicating how cell surface fluctuations and cell fluidity regulate early embryonic sorting in the ICM.

Given that pPrE and siACTN4 and siERM cells all display higher surface fluctuations inside of aggregates when they are mixed with their less active counterparts, we systematically measured the cell shape index for the more “active” cells in their respective aggregates. Strikingly, we observed in isolated ICMs that, even though pPrE and pEPI cells did not display significant differences in size (see STAR Methods), the mean shape index of the pPrE cells was significantly higher than in pEPI cells. This result indicates that pPrE cells are not only a more active but also a more fluid population of cells (Figure 7A). Furthermore, we also found that the shape index was higher in siACTN4 and siERM cells than in their less active counterparts (Figure 7B). We then found, using vertex model simulations, that inputting the measured differences in shape index in a simulated 3D tissue gave rise to demixing (Figure 7C). Although this mode of demixing arising from shape differences has been shown to be only local and partial (Sahu et al., 2020), the sorting in our 3D simulations occurs at the experimentally relevant length scales of the ICM.

Given recent work on monolayers with variable cell-cell tensions leading to tissue fluidization (Kim et al., 2021; Krajnc, 2020; Yamamoto et al., 2020), we next wondered if the fluid subtype in our model of demixing aggregates shows large surface tension variability. We therefore assessed, using vertex modeling, the variability in surface tensions (also called edge tensions in vertex models) that arise in a mixed population of more fluid (large shape index) and more solid (small shape index) cells. We found that the fluid cells do indeed have more variable surface tensions than the solid cells (Figure 7D, right). Moreover, they can display negative edge tensions (Figure 7D, left), which can arise in vertex models if adhesion forces are larger than tensile forces, and would lead to junctions tending to elongate rather than contract. A negative edge tension would tend to lead to bulging in the cells at junctions and potentially at free surfaces. Together, more variable surface tensions and the occurrence of negative tensions suggest a greater capacity for blebbing and surface fluctuations in general in more fluid cells (Figure 7D). This greater capacity for surface fluctuations provides an intriguing theoretical link between our two key phenomenological observables, cell fluidity and surface fluctuations.

We then reasoned that if there is a physical connection between cell shape and fluctuations, there ought to be a strong correlation in our cell data. To quantify this first in the ICM, we compared the variations in shape index (as a readout of fluctuations) with the shape index itself and found a strong correlation between the two (Figure 7E). Furthermore, consolidating all our data in ES cells and ICM, we found that the more “fluid” cells (upper half of the shape index distribution) displayed significantly higher fluctuations in shape index than the more “solid” cells (lower half of the shape index distribution) (Figure 7E bottom). Taking these data together with our simulation, we propose that there is a deep connection between the surface fluctuations we observe and cell fluidity. In summary, our data suggest that higher cell surface fluctuations are associated with higher cell fluidity, and that higher fluidity and surface fluctuations together lead to pPrE cells demixing from the more solid pEPI cell population.

## Discussion

There has been a growing consensus that spatial segregation of embryonic cell lineages is typically driven by cell-cell affinity asymmetries at cellular interfaces (Amack and Manning, 2012; Chan et al., 2017; Krieg et al., 2008; Maître et al., 2012, 2016), though a recent report found spatial segregation with no clear affinity asymmetries (Krens et al., 2017). Here, our simulations suggest that for cell sorting to occur within a time frame relevant to development, a high degree of affinity asymmetry is required, which is not necessarily achieved for all embryonic tissues, including EPI and PrE in the mouse blastocyst. For our analysis, we chose the most extreme possible value of the difference in cell-cell affinity from our measurements to run our simulations. Thus, though we cannot entirely exclude the possibility that the small differential cell-cell affinity we observed leads to an “outside bias” for the PrE lineage, none of our measurements support differential cell-cell affinity as a driving force of sorting. Therefore, we conclude that difference in cell-cell affinity alone do not explain why PrE cells sort to the outside.

Our simulations and experimental models strongly suggest that it is not only average levels of cell surface mechanical factors, but also their dynamics and concomitant cell surface fluctuations, that are important for sorting (Figure 7F), contrasting with classical models for the phase separation of binary cellular mixtures (Graner and Glazier, 1992) based on minimization of an effective energy. These cell surface fluctuations manifest differently depending on whether cells have a free surface or if they are immersed in the bulk of a multicellular aggregate. With a free surface, fluctuations primarily appear as blebs, but within the bulk they appear as shape fluctuations. It is likely that the many of the same dynamical mechanical instabilities are responsible for both these manifestations of surface fluctuations.

Our work suggests at least two areas of future work. First, how are cell surface fluctuations molecularly regulated? Our data imply that ERM-regulated effective membrane tension, a key regulator of cell surface fluctuations, could be a pivotal and previously overlooked player in tissue sorting. Indeed, changing ERM levels resulted in fluctuations that directly affected sorting both in cell aggregates (Figures 5D and 5E) and embryos (Figure 6). On the other hand, it is unlikely that surface fluctuations arise from the activity of a single mechanical regulator; indeed, it is likely that mechanical regulators such as ERM and ACTN4 are part of a larger network of mechanical regulators that control the level of surface fluctuations. Given that our data also suggest that the enhanced surface fluctuations in the PrE lineage are propelled by FGF signaling, it is plausible that the changes we see in mechanical factors are part of a larger network of changes in intracellular signaling connected to the emerging identity of the PrE lineage. Notably, ERMs are also involved in polarity establishment, and polarity has been shown to crucially contribute to PrE lineage specification and positioning (Bassalert et al., 2018; Gerbe et al., 2008; Saiz et al., 2013; Suzuki et al., 2002). At this point, how polarity is connected to a mechanical mechanism of sorting is unknown; nevertheless, there is extensive cross-talk between polarity signals and regulators of cell mechanics, such as Rho GTPases (Iden and Collard, 2008; Zhu et al., 2017). Thus, the increased surface fluctuations we observe might be the mechanical signature of nascent polarity. Importantly, however, the mechanical mechanism of sorting we propose does not require polarity. Instead, our model and experiments point to an initial sorting mechanism purely driven by differences in surface fluctuations and cell fluidity. Future work will be needed to disentangle the how different molecular players, including polarity factors and actin cortex regulators, control heterogeneities in membrane tension and the degree of cell fluidity in the developing embryo.

The second open question concerns the relationship between cell surface fluctuations and preferred cell shape (a classical measure of local tissue fluidity; Bi et al., 2015; Park et al., 2015). Though it is not yet possible to establish if there is a hierarchy between cell surface fluctuations and cell shape and fluidity, our work suggests that that preferred shape and fluctuations are likely intrinsically coupled. Indeed, our vertex model simulations suggest that the subtype with higher cell fluidity also has higher variations in cell surface tensions and can display negative surface tensions, which could favor cell surface deformations such as blebbing. Future studies could explore the relationship between fluctuations and cell shape and will require new modeling frameworks. Indeed, at this point, vertex modeling does not implement boundary conditions such as fluid interfaces in small systems such as small cell aggregates or the ICM (Sahu et al., 2020; Sussman et al., 2018); in contrast, such boundary conditions are readily implemented with particle-based models such as CS3D. Such an extension to vertex modeling could particularly help to understand how local demixing via differential cell shape/fluidity could result in a cell population specifically located at the boundary of an aggregate, with another more biased toward the aggregate core.

In summary, in this work, we propose a mechanism to explain spatial segregation in tissue based not on differences in static mechanical properties alone but relying instead on dynamical, non-equilibrium mechanisms. Such fluctuation-driven sorting could represent a broadly applicable sorting principle across species and tissues. Ultimately, our discovery that the differences in noise at cell surfaces and interfaces, or surface fluctuations, regulate the mechanical sorting of lineages in the mouse blastocyst provides insight into tissue self-organization in the early embryo. The fluctuation mechanism we propose here is likely linked to cell and tissue fluidization, which has been proposed to facilitate tissue morphogenesis. It will be interesting to investigate how cell surface dynamics influence other processes of self-organization across organisms, including tissue morphogenesis and tumorigenesis.

### Limitations of the study

We have identified several limitations in our study. First, the link between cell fluidity and fluctuations is correlative and thus currently somewhat speculative. Developing a means to incorporate tensional fluctuations will help to clarify this link in the future. Second, we have not yet identified a molecular mechanism to explain how the differences in cell surface fluctuations between pPrE and pEPI cells arise. Likewise, we have not identified how FGF signaling leads to greater surface fluctuations. Finally, given that perturbing cell surface fluctuations directly in the embryo is likely to influence lineage choice, we used chimeras incorporating embryonic stem cells as an experimental model to show that cell surface fluctuations influence sorting *in vivo*. Even though embryonic stem cells will contribute to embryonic tissue after injection, we are not perturbing cells native to the embryo and are thus not precisely modeling the cell-sorting process *in vivo*. All of these limitations could be addressed with their own studies and point toward interesting future work that would extend our findings.

## STAR★Methods

### Key resources table

**Table:** 

REAGENT or RESOURCE	SOURCE	IDENTIFIER
**Antibodies**		

Rat anti-SOX2	eBioscience	Cat#14-9811-80; RRID: AB_11219070
Rat anti-NANOG	eBioscience	Cat#14-5761-80; RRID: AB_763613
Goat anti-SOX7	R&D Systems	Cat#AF2766; RRID: AB_2196241
Goat anti-SOX17	R&D Systems	Cat#AF1924; RRID: AB_355060
Rabbit anti-GATA4	Santa Cruz	Cat#sc9053; RRID: AB_2247396
Mouse anti-CDX2	Biogenex	Cat#MU392A; RRID: AB_2650531
Rabbit anti-Phospho-Ezrin (Thr567)/Radixin (Thr564)/Moesin (Thr558) (48G2) [pERM]	Cell Signaling	Cat#3141; RRID:AB_10560513
Rabbit anti-Phospho-Ezrin (Thr567)/Radixin (Thr564)/Moesin (Thr558) (41A3) [pERM]	Cell Signaling	Cat#3149; RRID:AB_823497
Rabbit anti-Phospho-p44/42 MAPK (Erk1/2) (Thr202/Tyr204) [pERK]	Cell Signaling	Cat#4370; RRID:AB_2315112
Rabbit anti-Phospho-Myosin Light Chain 2 (Ser19) [pMRLC]	Cell Signaling	Cat#3671S; RRID:AB_330248
Donkey anti-rabbit IgG H&L (Alexa Fluor®488)	Thermo Fisher Scientific	Cat#A-21432; RRID: AB_2535853
Donkey anti-goat IgG H&L (Alexa Fluor®555)	Thermo Fisher Scientific	Cat#A-21206; RRID: AB_2535792
Donkey anti-mouse IgG H&L (Alexa Fluor®555)	Thermo Fisher Scientific	Cat#A-31570; RRID: AB_2536180
Donkey anti-goat IgG H&L (Alexa Fluor®488)	abcam	Cat#ab150129; RRID: AB_2687506
Chicken anti-rat IgG H&L (Alexa Fluor® 647)	Thermo Fisher Scientific	Cat#A-21472; RRID: AB_2535875
Alexa Fluor™ 647 Phalloidin	Thermo Fisher Scientific	Cat#A22287; RRID: AB_2620155

**Chemicals, peptides, and recombinant proteins**		

KSOM	Millipore	Cat#MR-106-D
Blast	Origio	Cat#83060010
M2	Sigma	Cat#M7176
mineral oil	Sigma	Cat#M8410 (Batch: MKBW2313V, MLBV5961V)
Tyrode’s solution, Acidic liquid	Sigma	Cat#T1788
anti-mouse serum	Sigma	Cat#M5905
rat serum	prepared in-house	N/A
Accutase	PAA	Cat#L11-007
Trypisin	Invitrogen	Cat#25050030
chicken serum	Sigma	Cat#C5405
N2 (Batch tested)	prepared in-house	N/A
B27 (Batch tested)	Thermo Fisher Scientific	Cat#17504-044
L-glutamine	Thermo Fisher Scientific	Cat#25030024
Dulbecco’s Modified Eagle’s Medium/Nutrient Mixture F-12 Ham (DMEM/F12)	Sigma	Cat#D6421
Neurobasal (Batch tested)	Gibco™	Cat#21103049
Poly-D-lysine	Millipore	Cat#A-003-E
FGF2	prepared in-house	N/A
PD0325901	abcr	Cat#AB 253775
CHIR99021	abcr	Cat#AB 253776
CellMask Deep Red Plasma membrane Stain	Thermo Fisher Scientific	Cat#C10046
CellMask Orange Plasma membrane Stain	Thermo Fisher Scientific	Cat#C10045
CellMask Green Plasma membrane Stain	Thermo Fisher Scientific	Cat#C37608
Cell-Tak cell and tissue adhesive	Corning	Cat#354240
4',6-diamidino-2-phenylindole (DAPI)	Invitrogen	Cat#D1306
DRAQ 5™ Fluorescent prove solution	Thermo Fisher Scientific	Cat#62254
Hoechst 33342	Thermo Fisher Scientific	Cat#H3570
Alexa Fluor™ 647 Phalloidin	Thermo Fisher Scientific	Cat#A22287; RRID: AB_2620155
Alexa Fluor™ 488 Phalloidin	Thermo Fisher Scientific	Cat#A2315147; RRID: AB_2315147
gelatine from procine skin	Sigma	Cat#G1890
leukaemia inhibitory factor (LIF)	prepared in-house	N/A
Doxycyline (Dox)	Sigma	Cat#D9891
fetal bovine serum (FBS)	GE Healthcare Life Sciences,	Cat#SV30160.02
Glasgow’s minimum essential medium (GMEM)	Sigma	Cat#G5154
MEM non-essential amino acids (NEAA)	Sigma	Cat#M7145
sodium pyruvate	Sigma	Cat#S8636
In-Fusion® HD Cloning Kit	Clontech	Cat#639648
lipofectamin 2000 Transfection Reagent	Thermo Fisher Scientific	Cat#11668027
Geneticin™ Selective Antibiotic (G418 Sulfate)	Thermo Fisher Scientific	Cat#10131019

**Critical commercial assays**		

TaqMan Fast Universal Master Mix and TaqMan Gene Expression assays	Applied Biosystems	Cat#4366072
Mouse GAPD (GAPDH) endogenous control	Applied Biosystems	Cat#4352339E
SuperScript™ II Reverse Transcriptase	Invitrogen	Cat#18064022

**Deposited data**		

Single cell RNA seq data	This study	GEO accession GSE148462

**Experimental models: Cell lines**		

Mouse ES cells: E14Tg2a (E14)	(Hooper et al., 1987)	N/A
Mouse ES cells: Rex1-GFPd/Gap43-mCherry (Gap43-mCherry)	(Strawbridge et al., 2020)	NA
Mouse ES cells: R26-Confetti (Confetti)	Derived in house from a R26-Confetti mouse embryo	N/A

**Experimental models: Organisms/strains**		

Mouse: CD1	Charles River Laboratory	Strain Code 022
Mouse: C57BL/6JxCBA/J (F1 hybrid)	Charles River Laboratory	Strain Code 616
Mouse: *PdgfraH2B-GFP/+*	(Hamilton et al., 2003)	N/A
Mouse: mTmG (Gt(ROSA)26Sortm4(ACTB−tdTomato, −EGFP)Luo) (mTmG)	(Muzumdar et al., 2007)	N/A

**Oligonucleotides**		

SMARTpool siGENOME Mouse Ezrin	Dharmacon	Cat#M-046568-01-0005
SMARTpool siGENOME Mouse Moesin	Dharmacon	Cat#M-044428-01-0005
SMARTpool siGENOME Mouse Radixin	Dharmacon	Cat#M-047230-01-0005
SMARTpool siGENOME Mouse Actinin4	Dharmacon	Cat#M-049970-00-0005
control siRNA	Dharmacon	Cat#D-001210-02-05

**Recombinant DNA**		

pPB-CMV-HA-pA-IN	Prof Hitoshi Niwa (Kumamoto University, Kumamoto, Japan)	N/A
pPB-Tet-Ezrin-T567D	This study	N/A
pPB-CAG-rtTA-IN	Smith lab (Cambridge Stem Cell Institute, Cambridge, UK)	Addgene Plasmid #60612
pPy-CGA-Pbase	Smith lab (Cambridge Stem Cell Institute, Cambridge, UK)	N/A
human Ezrin_T567D cDNA	Prof G. Charass (UCL, London, UK)	N/A

**Software and algorithms**		

Fiji	(Schindelin et al., 2012)	https://www.r-project.org
Andor IQ Software	Andor Technology	http://www.andor.com/scientific-software/iq-live-cell-imaging-software
Matlab	MathWorks	https://www.mathworks.com/products/matlab.html
Prism 7	Graphpad software, Inc	https://www.graphpad.com/
htseq-count	(Anders et al., 2015)	https://htseq.readthedocs.io/en/master/
DESeq2	(Love et al., 2014)	https://bioconductor.org/packages/release/bioc/html/DESeq2.html
Sincell	(Juliá et al., 2015)	http://bioconductor.org/packages/release/bioc/html/sincell.html
FactoMineR	(Lê et al., 2008)	http://factominer.free.fr/
R monocle package	(Trapnell et al., 2014)	https://cole-trapnell-lab.github.io/monocle3/
Ensembl 87	N/A	https://www.ensembl.org/index.html

**Other**		

Mouse *Ezrin* TaqMan Probe	Thermo Fisher Scientific	Cat#Mm00447761_m1
Mouse *Moesin* TaqMan Probe	Thermo Fisher Scientific	Cat#Mm00447889_m1
Mouse *Radixin* TaqMan Probe	Thermo Fisher Scientific	Cat#Mm01177363_m1
Mouse *a-actinin4* TaqMan Probe	Thermo Fisher Scientific	Cat#Mm00502489_m1

### Resource availability

#### Lead contact

Further information and requests for resources and reagents should be directed to and will be fulfilled by the lead contact, Kevin J Chalut (kc370@cam.ac.uk).

#### Materials availability

This study did not generate new unique reagents.

### Experimental model and subject details

#### Mouse strains and embryo collection

Mice used were intercrosses of *Pdgfra*^*H2B-GFP/+*^ (Hamilton et al., 2003), in which a cassette containing human H2B fused to enhanced green protein (H2B-GFP) was targeted to the *Pdgfra* locus, and first filial generation (F1) hybrids (C57BL/6JxCBA/J) (Charles River), homozygous mTmG [Gt(ROSA)26Sor^*tm4(ACTB−tdTomato, −EGFP)Luo*^, mTmG^*+/+*^] (Muzumdar et al., 2007) or CD-1 (Charles River). CD-1 embryos were used for the injection experiments. All embryos used in this study were obtained from natural mating. Embryo staging was based on the assumption that, on average, mating occurred at midnight so that at midday, the embryos were assigned E0.5. Embryos were flushed at the relevant stages from oviducts (eight-cell stage embryos) or uterine horns (blastocysts) using flushing and holding media (M2, Sigma). PrE cells can be visualised with *Pdgfra*^*H2B-GFP/+*^ reporter which we used as additional criteria to classify embryo stages (Grabarek et al., 2012; Plusa et al., 2008). EPI and PrE have not segregated at E3.5 - E3.75 stages. EPI and PrE have segregated at E4.0 - E4.5. GFP positive PrE were clearly seen to form one layer faced with a blastocoel. The sex of embryos and the ages of mice using mating were not concerned in this study. The mice were maintained in a state-of-the-art biofacility with daily health checks carried out by dedicated trained staff. The mice were maintained on a lighting regime of 12:12 hours light:dark with food and water supplied ad libitum. This research has been regulated under the Animals (Scientific Procedures) Act 1986 Amendment Regulations 2012 following ethical review by the University of Cambridge Animal Welfare and Ethical Review Body (AWERB). Use of animals in this project was approved by the ethical review committee for the University of Cambridge, and relevant Home Office licences (Project licence No. 80/2597 and No. P76777883) are in place.

#### Cell cultures

ES cell lines are listed in the key resources table. ES cells were routinely maintained on 0.1% gelatine (Sigma)-coated 6-well plates (Falcon) in 2i+LIF media (Ying et al., 2008), which contains N2B27 medium supplemented with 1 μM PD03 (abcr) and 3 μM CHIR99021 with 10 ng/ml LIF (in-house). Cells were passaged every three days, using Accutase disassociation (PAA). N2B27 media were prepared as described (Ying et al., 2008). Briefly, 1:1 Dulbecco’s Modified Eagle’s Medium/Nutrient Mixture F-12 Ham (DMEM/F-12; Sigma) and Neurobasal media (Gibco), N2 (in-house) and B27 (Thermo Fisher Scientific) additives, 2 mM L-glutamine (Thermo Fisher Scientific), and 100 mM 2-mercaptoethanol (Sigma) were supplemented. Cells were cultured without antibiotics and tested negative for mycoplasma by periodic PCR screening.

#### Generation of H2B-BFP, tdTomato and Dox-inducible EzrinT567D (CA-EZR) ES cells

tdTomato ES cells were generated from the embryo crossed Gt(Rosa)26Sor^tm9(CAG-tdTomato)Hze^ mice (JAX#007909 [Madisen et al., 2010]) with R26Cre^ER^ mice (JAX#004847 [Badea et al., 2003]). 500 nM 4-Hydroxytamoxifen (Sigma) was added to the ES cells and tdTomato-positive ES cells were expanded.

For H2B-BFP ES cells generation, 0.8 μg of pPB-CAG-H2B-BFP-IRES-Neo (kindly gifted by M Kinoshita) and 0.4 μg of pPy-CGA-PBase were transfected into E14Tg2A (E14) ES cells using Lipofectamine 2000 (Thermo Fisher Scientific). Following the drug selection with 400 μg/ml G418 (Thermo Fisher Scientific), BFP-positive colonies were picked and expanded.

Human EzrinT567D (constitutive active form of Ezrin; CA-EZR) cDNAs were a kind gift from G. Charass. They were inserted into pPB-CMV-HA-pA-IRES-Neo (kindly gifted by H. Niwa) using In-Fusion (Clontech) to generate pPB-Tet-CA-EZR. 0.8 μg of pPB-Tet- CA-EZR was transfected with 0.8 μg of pPB-CAG-rtTA-IRES-Neo (kindly gifted by A Smith [Addgene plasmid #60612] [Takashima et al., 2014]) and 0.4 μg of pPy-CGA-PBase using Lipofectamine 2000 into E14 ES cells. The cells were harvested from a 0.1% gelatine-coated 6-well plate in 2i+LIF. After 48 hours, 400 μg/ml G418 was added to ES cells and colonies were selected. After one week of G418 selection, clones were manually picked, dissociated, then split into a 96-well plate (Corning). ES cells were cultured with 2i+LIF in the presence or absence of 1 μg/ml Dox (Sigma). Clones that with no mCherry signal in the absence of Dox and high mCherry signal in the presence of Dox were chosen by eyes.

### Methods details

#### Isolation of ICMs from embryos, and single-cell dissociation of ICMs

Embryo and cell manipulations were carried out under a dissecting microscope (Leica Microsystems). The zona pellucida was removed using acid Tyrode’s solution (Sigma). Blastocysts from E3.5-E4.5 were subjected to immunosurgery as previously described (Solter and Knowles, 1975). In brief, blastocysts were incubated for 45-60 minutes in a 1:5 dilution of anti-mouse rabbit serum (Sigma) in N2B27, washed in N2B27 and further incubated for 30-60 minutes in a 1:5 dilution of rat serum (in-house) in N2B27 for the complement reaction. The ICM was subsequently cleaned from residual trophectoderm with a narrowly fitting glass pipette. Single-cell dissociation of ICMs was performed in a 1:1 mixture of Accutase and 0.025% trypsin (Invitrogen) plus 1% chick serum (Sigma). Cells were dissociated by repetitive using blunted microcapillaries (Global Scientific or Harvard apparatus) and washed in Blast (Origio) or N2B27.

#### Embryo and ICM culture

Embryos and isolated ICMs were cultured in Blast, KSOM (Millipore) or N2B27 in an organ culture dish (Falcon) culture or in single-drop cultures under mineral oil (Sigma) in a humidified incubator at 37^°^C with 5% CO_2_. Embryo culture media were buffered in the incubation chamber for at least 30 minutes before embryos and ICM culture.

#### Live imaging of isolated ICMs

Isolated ICMs were transferred to an embryo immobilization chip (Dolomite Centre Ltd). The spinning disk microscope (Andor Revolution XD System [ANDOR] with a Nikon Eclipse Ti microscope [Nikon]) was used for taking images. 17 z-stacks per time step every 30 minutes were taken, with three channels (488 nm excitation for *Pdgfra*^*H2B-GFP/+*^ reporter, 561 nm excitation for membrane [mTmG] and bright field). An Andor 85 camera recorded images with magnification through a CFI Plan Fluor ×40/1.3 oil objective (Nikon) with Cargille microscope immersion oil (Cargille Labs). Each experiment was set up using Andor IQ Software. Each image collected data in 502 × 501 (width × height) pixels. The microscope is equipped with an incubation chamber to keep the sample at 37°C and 7% CO_2_. Images were processed using Fiji (Schindelin et al., 2012).

#### Measurements of isolated EPI and PrE size

EPI and PrE cells’ sizes were measured using the images from the middle plane of the cells. For E3.5 cells, six EPI and six PrE cells were measured. For 3.75 cells, 21 pEPI and 22 pPrE cells were measured. For E4.5 cells, ten EPI and eight PrE cells were measured.

#### Immunofluorescence staining

Embryos and isolated ICMs were fixed with 4% paraformaldehyde (PFA; Thermo Fisher Scientific) in Phosphate buffered saline (PBS; Sigma) at room temperature for 15 minutes. Then, the samples were rinsed in PBS containing 3 mg/ml polyvinylpyrrolidone (PBS/PVP; Sigma), permeabilised with PBS/PVP containing 0.25% Triton X-100 (Thermo Fisher Scientific) for 30 minutes. Blocking was performed with an embryo blocking buffer comprising PBS containing 0.1% bovine serum albumin (BSA; Sigma), 0.01% Tween20 (Sigma) and 2% donkey serum (Sigma) at 4^°^C for 2-3 hours. Primary antibodies were diluted in an embryo blocking buffer, and samples were incubated with the antibody solution at 4^°^C overnight. They were rinsed three times in an embryo blocking buffer for 15 minutes ∼ each. Secondary antibodies were diluted in an embryo blocking buffer with or without 500 ng/ml 4',6-diamidino-2-phenylindole (DAPI; Invitrogen), and samples were incubated in the appropriate antibody solution at room temperature for one hour in the dark. They were rinsed three times in an embryo blocking buffer for 15 minutes ∼ each, then transferred in small drops of blocking buffer on a poly-D-lysine (PDL, Millipore) coated glass-bottom dish under the mineral oil and taken images. For the coating, the dishes were coated with small drops of 50 μg/ml PDL for at least one hour at room temperature. The drops were washed three times with the media used for imaging and covered with mineral oil. Otherwise, the samples were incubated briefly in increasing concentrations of Vectashield (Vector Laboratories) before mounting on glass slides in small drops of concentrated Vectashield. Subsequently, coverslips with Vaseline spacer were mounted and sealed with nail varnish. Whole staining process was performed on Pyrex 9 depression spot plate (Corning).

For pERM staining, pEPI and pPrE cells seeded on a PDL-coated 10-well slide glass (TF1006, MTSUNAMI) were fixed with 4% PFA in cytoskeletal stabilizing buffer (CSB; 10 mM MES pH6.1, 138 mM KCl, 3 mM MgCl_2_, 2 mM EGTA) containing 4.5% w/v sucrose (Sigma) and 0.2% Triton X-100 at 37^°^C for six minutes. Subsequently, the samples were fixed with 4% PFA in CSB containing 4.5% w/v sucrose at 37^°^C for 14 minutes. The fixation buffers were pre-warmed at 37^°^C before use. Then, the samples were rinsed in PBS for twice, permeabilised with PBS containing 0.1% Triton-X at room temperature for ten minutes. Blocking was performed with a buffer comprising PBS containing 2% FBS, 2% BSA and 0.1% Triton-X at room temperature for 45 minutes. Primary antibodies were diluted in blocking buffer, and samples were rinsed with the appropriate antibody solution once and incubated with the antibody solution at 4^°^C overnight. They were rinsed five times using PBS containing 0.1% Triton-X for five minutes ∼ each. Secondary antibodies were diluted in blocking buffer, and samples were incubated in the appropriate antibody solution at room temperature for one hour in the dark. They were rinsed five times in PBS containing 0.1% Triton-X for five minutes ∼ each. 10 μl of Vectashield was added to each well, and coverslips were mounted and sealed with nail varnish. For pMRLC staining, cells were fixed and stained in a similar manner to pERM staining. PBS containing 1% BSA and 10% donkey serum was utilised for blocking buffer. Primary and secondary antibodies were listed in key resources table.

#### Imaging

For embryos, isolated ICMs, ICM cells, ES cells imaging, samples were transferred to the drops on PDL-coated glass-bottom dishes and taken images using a Leica TCS SP5, Leica Stellaris or ZEISS LSM980 confocal microscope. For live imaging of ICM aggregates, doublets of ICM cells and ES cells, SP5 was used with an incubation chamber to keep the sample at 37°C and 7% CO_2_. For the quantification of fluorescence in single cells, a set of experiment images were acquired using the same microscope with the same setting on the same day. Images were then segmented to isolate the signal and mean intensity was found using Fiji.

#### cDNA amplification and synthesis from single cells

Three E3.75 *Pdgfra*^*H2B-GFP/+*^ positive embryos obtained from intercrossing of *Pdgfra*^*H2B-GFP/+*^ and F1 hybrids were used. We collected embryos, which had salt-and-pepper GFP positive cells distribution in ICM and proper GFP intensity. ICM cells were dissociated from isolated ICM by immunosurgery. Dissociated single cells were transferred immediately into Smart-Seq2 single-cell lysis buffer and immediately frozen on dry ice. Smart-Seq2 library was prepared as originally described (Picelli et al., 2014). Briefly, 8-well strips containing isolated single nuclei in lysis buffer were thawed, and reverse transcription using Superscript II (Thermo Fisher Scientific) with olig-dT30-VN and TSO primers and PCR using KAPA Hifi HotStart ReadyMix (Kapa) with ISPCR primer. Following RT-PCR, clean up with Agencourt AMPure XP beads (Beckman Coulter) was carried out. The Nextera XT DNA library prep kit (Illumina) was used for subsequent sample preparation. The samples were subjected to a tagmentation reaction, indexing and PCR amplified. Libraries were then mixed and purified with Agencourt AMPure XP beads. Ready DNA libraries were quality controlled using Qubit Fluorometer (Thermo Fisher Scientific) and Bioanalyzer (Agilent Technologies). The samples were sequenced on the Illumina HiSeq2000 platform (150 base, paired end).

#### RNA-seq data processing

Sequencing data of single-cell mouse embryo profiling study (accession SRP110669 [Mohammed et al., 2017]) was downloaded from the European Nucleotide Archive (Toribio et al., 2017). Mus musculus GRCm38.87 gene annotation was used together with mm10 genome version. Alignments to gene loci were quantified with htseq-count (Anders et al., 2015) based on annotation from Ensembl 87. Sequencing libraries with fewer than 500K mapped reads were excluded from subsequent analyses. Read distribution bias across gene bodies was computed as the ratio between the total read spanning the 50th to the 100th percentile of gene length, and those between the first and 49th. Samples with ratio >1.5 were not considered further. Stage-specific outliers were screened by principal component analysis.

#### Transcriptome analysis

Principal component and cluster analyses were performed based on log_2_ fragments per kilobase of exon per million mapped fragments (log_2_FPKM) values computed with the Bioconductor packages *DESeq2* (Love et al., 2014), *Sincell* (Juliá et al., 2015) or *FactoMineR* in addition to custom scripts. Differential expression analysis was performed with scde (Kharchenko et al., 2014), which fits individual error models for the assessment of differential expression between sample groups. Pseudotimes were computed using R monocle package (Trapnell et al., 2014). For global analyses, genes that registered zero counts in all single-cell samples in a given comparison were omitted. Euclidean distance and average agglomeration methods were used for cluster analyses. Expression data are available upon request. Ensembl 87 annotation was used to download specific actin cytoskeletal genes using biological process name as a keyword.

#### Selection of high-variability genes

Gene exhibiting the greatest expression variability (and thus contributing substantial discriminatory power) were identified by fitting a non-linear regression curve between average log_2_ FPKM and the square of the coefficient of variation. Thresholds were applied along the *x*-axis (average log_2_ FPKM) and *y*-axis (log squared coefficient of variation [CV^2^]) to identify the most variable genes. As actin-cytoskeleton-related genes, we selected 6899 genes (Table S1). Amongst them, 152 genes were highly modulated in ICM cells through E3.5 to E4.5 blastocysts and use these genes for actin-cytoskeleton related genes principal component and cluster analyses (Figure 1E; Table S1).

#### ICM cell migration assay

E3.75 *Pdgfra*^*H2B-GFP/+*^ positive embryos obtained from intercrossing of *Pdgfra*^*H2B-GFP/+*^ and CD-1 or F1 hybrid were used. Isolated single ICM cells in Blast medium containing 1:10000 CellMask Orange (Life Technologies) were loaded into the BSA coated polydimethylsiloxane (PDMS) confinement devices with a fixed roof height of 8 μm, 9 μm or 10 μm. Confinement devices were designed to restrict cells between two glass plates, trapping the cells in the *z*-direction but allowing free movement in *x*- and *y*-direction, as first demonstrated in (Le Berre et al., 2014). To adapt the device for the use with very small cell numbers, we modified confinement channels as described in Heuzé et al. (2011) by replacing the channels with pillars to create a constant, well-defined roof height. Live imaging of the cells was performed with a 6x silicon objective (UPLSAPO60XS, Olympus) on an inverted microscope (Olympus FV1200) equipped with a humidified chamber at 37°C and 5% CO_2_. The bright field, GFP, and CellMask images of the cells were taken every one, two or three minutes for up to ten hours.

#### Surface tension measurement

##### Cell preparation

E3.75 *Pdgfra*^*H2B-GFP/+*^ embryos obtained from intercrossing of *Pdgfra*^*H2B-GFP/+*^ and F1 hybrids were used. Isolated ICM cells were transferred to single-drop of N2B27 in a glass-bottom dish (FluoroDish, World Precision Instruments) and incubated for 30 minutes at 37°C and 5% CO_2_. 2 ml M2 in the presence of 0.01% CellMask Deep Red Plasma membrane Stain (Thermo Fisher Scientific) was added to the dish. The mean GFP intensity of *Pdgfra*^*H2B-GFP*^ was used to classify ICM cells’ lineage and cell cycle stage. Mitotic cells and dead cells were excluded from the analysis. Samples were measured for no longer than two hours.

For ES cells, H2B-BFP or CA-EZR cells were seeded on 0.1% gelatine-coated 6-well plates in 2i+LIF at 2.0×10^4^ cells, and the media was changed to 2i+LIF with or without 1μg Dox following day. The following day, cells were detached using Accutase and suspended in 2i+LIF. Suspended cells were centrifuged at 1400 rpm for three minutes, pelleted, re-suspended in 2i+LIF in the presence of 0.01% CellMask Green Plasma Membrane Stain (Thermo Fisher Scientific) and added to a PDL-coated glass-bottom dish. The cells were incubated for 30 minutes at 37°C and 5% CO_2_. Mitotic cells and dead cells were excluded from the analysis. Samples were measured for no longer than 50 minutes.

##### Experimental setup

pEPI and pPrE tension measurements were performed using a JPK CellHesion (JPK Instruments) mounted on an IX81 inverted confocal microscope (Olympus). Tipless silicon cantilevers (ARROW-TL1Au-50) were chosen with a nominal spring constant of 0.03 N/m. Sensitivity was calibrated by acquiring a force curve on a glass coverslip. Spring constant was calibrated by the thermal noise fluctuation method. *Z*-length parameter and setpoint force were set at 30 μm and 10 nN, respectively. Constant height mode was selected. The measurement was carried on by lowering the tipless cantilever onto an empty area next to a target cell. Once the cantilever retracted (by roughly 30 μm), it was positioned above the target cell and run a compression for 200 seconds. During the constant height compression, the force acting on the cantilever was recorded. After initial force relaxation, the resulting force value was used to extract surface tension. A confocal stack was acquired using an Olympus UPLANSAPO ×60/1.35 NA oil immersion objective (Olympus).

ES cells tension measurements were performed using a JPK CellHesion 200 (Bruker Corporation) and a DSD2 Differential Spinning Disk (Andor) both mounted on a DMi8 inverted microscope (Leica). Tipless silicon cantilevers (ARROW-TL1-50) were chosen with a nominal spring constant of 0.03 N/m. Sensitivity was calibrated by acquiring a force curve on glass. Spring constant was calibrated by the thermal noise fluctuation method. *Z*-length parameter and setpoint force were set at 80 μm and 4 nN, respectively. Constant height mode was selected. The measurement was carried on by lowering the tipless cantilever onto an empty area next to a target cell. Once the cantilever retracted (by roughly 80 μm), it was positioned above the target cell and a compression was run for 50 seconds. During the constant height compression, the force acting on the cantilever was recorded. After initial force relaxation, the resulting force value was used to extract surface tension. A confocal stack was acquired using a ×40/1.1 NA water immersion objective (Leica).

##### Analysis

The calculation of cortex tension (T) is based on Fischer-Friedrich et al. (2014) (Equation 2). Briefly, neglecting the angle of the cantilever with respect to the dish (∼8°) for pPrE and pEPI tension measurements, to the dish (∼10°) for pPrE and pEPI tension measurements and assuming negligible adhesion between cell, dish and cantilever, the force balance at the contact point reads:

(Equation 2) $$\text{T}=\frac{\text{F}\left(\frac{r_{\text{mid}}^{2}}{r_{\text{c}}^{2}}-1\right)}{2\text{π}r_{\text{mid}}}$$

where *r*_mid_ is the radius of maximum cross-sectional area of the selected cell, *r*_c_ is the radius of contact area between cell and cantilever and *F* is the resulting force exerted by the cell on the cantilever. To avoid errors due to direct measurement of *r*_c_, the contact radius was calculated using the following Equation 3 (Stewart et al., 2012):

(Equation 3) $$A_{\text{c}}=A_{\text{mid}}-\left(\frac{\text{π}}{4}\right){\text{h}}_{\text{cell}}^{2}$$

where *A*_c_ is the contact area between cell and cantilever, *A*_mid_ is the cell maximum cross-sectional area and *h*_cell_ is the cell height. *h*_cell_ was calculated as described (Stewart et al., 2012) from the radius, force and cantilever height during compression. The cantilever height during compression was obtained by subtracting the cantilever height difference on glass and the cantilever height difference during cell compression. For pPrE and pEPI tension measurements, *h*_cell_ values were confirmed by CellMask™ Deep Red membrane confocal stack reconstruction (corrected for optical aberration) (Chugh et al., 2017; Diaspro et al., 2002; Hell et al., 1993). For the ES cell analysis, some of the shapes were difficult to approximate leading to difficulties estimating the radius, leading in turn to abnormally high surface tension measurements. We performed a boxplot analysis with a whisker size of 1.5 to remove those outliers, which were scattered across all groups and represented less than 10% of the overall measurements.

#### Doublet formation

##### Forming doublets

E3.75 *Pdgfra*^*H2B-GFP/+*^ positive embryos obtained from intercrossing of *Pdgfra*^*H2B-GFP/+*^ and F1 hybrids were used. Two isolated single ICM cells were put together by gently blowing the surrounding medium through a microcapillary in the micro drop of Blast under the mineral oil and incubated for 30 minutes at 37°C and 5% CO_2_. When two isolated ICM cells come into contact, the contact grows until equilibrium is attained. Doublets were transferred in Blast drop in the presence of 0.01% CellMask™ Deep Red Plasma membrane Stain to visualise membrane under the mineral oil in a glass-bottom dish (MatTek) coated with PDL (see Figure S2I). Before transferring doublets to Blast drop, the drops under mineral oil were buffered in the incubation chamber for at least 30 minutes. Confocal images were acquired using a Leica TCS SP5 (Leica Microsystems) confocal microscope. Optical section thickness was 0.99 μm. A HC PL APO 40×/1.30 Oil CS2 (Leica) with immersion oil (Leica) was used. Whole doublet images from bottom to top were taken with three channels (488 nm excitation for *Pdgfra*^H2B-GFP^ reporter, 647 nm excitation for membrane [CellMask™ Deep Red Plasma membrane Stain] and bright field). The microscope is equipped with an incubation chamber to keep the sample at 37°C and 7% CO_2_.

##### Forming doublets from ES cells

CA-EZR-IRES-mCherry were seeded on 0.1% gelatine coated 6-well plates for 24 hours in N2B27+2i+LIF at a density of 2 × 10^4^ cells/cm^2^ before addition of 1μg/ml Dox in N2B27+2i+LIF to one well whilst leaving another well without Dox as control for further 24 hours. The following day, cells were dissociated into a single-cell suspension using Accutase and suspended in 500 μl each culture media with 0.01% CellMask™ Green Plasma membrane Stain and Hoechst 33342 (Thermo Fisher Scientific). The cell suspension was seeded on PDL-coated dishes and incubated at 37^°^C 5% CO_2_ for 30 minutes to allow doublet formation before live imaging.

##### Measuring contact size or contact angle measurement in doublets

The external contact angles at the middle section of the doublets were measured by using the angle tool of Fiji. The average of both sides of the external contact angles was used as the external contact angle *θ*_e_. The contact size was measured by drawling a segment along the waist of the doublet and measuring the length of the segment using Fiji. The mean GFP intensity of *Pdgfra*^*H2B-GFP*^ was quantified using Fiji and used to classify the lineage of each ICM cell. The top and bottom 40% of cells with strong GFP intensities (GFP^high^ cells and GFP^low^ cells) were considered as pPrE cells and pEPI cells, respectively, and used for this analysis. The doublets that were not horizontal to the dish, mitotic cells, dead cells and blebbing cells at the interface were excluded from the analysis.

#### CS3D method: Modeling surface fluctuations and blebbing

Simulation results in this work were produced by the cell sorting in 3D (CS3D, http://github.com/chris-revell/SEM) model. The full explanation and theoretical foundations of this model are contained in references (Revell, 2018; Revell et al., 2019). Briefly, CS3D is an extension of the Subcellular Element Method (Newman, 2007), a force-based technique for modelling the development of multicellular tissues. It allows us to study the effects of complex inter- and intra-cell features on tissue-scale dynamics (Sandersius and Newman, 2008). Each individual cell is modelled as a group of infinitesimal elements, interacting via nearest-neighbour forces (Figure 1 of Revell et al., 2019) defined by Morse potentials (Morse, 1929). Nearest-neighbour elements of different cells interact by the same mechanism. This model produces a fine-grained representation of multicellular systems incorporating both inter- and intra-cellular mechanisms. We implemented a simple algorithm for identifying boundary elements in each cell (Revell, 2018) and used a Delaunay triangulation (Delaunay, 1934; Renka, 1997) over this set of elements to define a nearest neighbour network across the cell boundary. By applying a constant force between these neighbouring elements in the triangulation, we modelled cell cortical tension. The magnitude of tension forces defined within the Delaunay triangulation can vary locally across the cell surface at interfaces with surfaces of other cells. We define a cell-cell interface by identifying and labeling the cortex elements that share adhesive inter-cell interactions with cortex elements of another cell (Figure 1 of Revell et al., 2019).

The theoretical foundations of CS3D are explained in reference (Revell et al., 2019), particularly section A of the Supplement of that reference, and the parameters used in the simulation are listed in the Tables in section B of the Supplement for reference (Revell et al., 2019). Briefly, parameters such as cell-cell adhesion, intefacial tension and cell stiffness are relative measurements, chosen with reference to ‘stiffness_factor’ listed in the table in Section B of reference (Revell et al., 2019). The ‘stiffness_factor’ was parameterized in the initial subcellular element method and was validated by comparison to the viscoelasticity of living cells (Sandersius and Newman, 2008). We parameterized the surface mechanics in CS3D by ensuring that the contact area of a doublet scaled with cell-cell affinity in agreement with the linear force balance model demonstrated in reference (Maître et al., 2012). In this way, for the modelling results of this paper, we chose a cortical tension of surface tensions (*γ*_*cm*_) *= 1.4* (as in Figure 2A) and an adhesion strength *A*_*M*_ *= 0.3*. The full explanation and justification for the variables and parameters we used in the model are in reference (Revell et al., 2019), particularly Figure 2. All other parameters tested for the purposes of the current work, including affinity parameter and surface fluctuations, are explicitly stated within the text and/or figures.

Importantly, in Figure 4 of Revell et al. (2019), reproduced at right, we show that analysis of doublet contact areas between cell types is a nearly perfect predictor of sorting in a multicellular aggregate, regardless of the underlying forces that gives rise to that contact area. Cell-cell affinity, as measured by contact area, is a result of a balance between cell-cell adhesion, cortical tension, and interfacial cortical tension. A number of experimental and theoretical studies (Amack and Manning, 2012; Chan et al., 2017; Maître et al., 2012, 2015) have shown that cell-cell affinity, not adhesion, cortical tension, or interfacial tension, is the relevant mechanical parameter to consider when studying sorting. To produce the results at right, we used the same CS3D model utilised in the current manuscript to study the relationship between cell-cell affinity and sorting. We varied cell-cell adhesion, interfacial tension and cortical tension differently across two different types of cells. Then a virtual doublet was modelled for each type of cell, and the contact area was found. The x-axis at right represents the absolute value of the difference in contact area for each cell. Then a virtual aggregate of 10 cells was modelled with the two types of cells using the same set of mechanical parameters used for the virtual doublet, then evolved to 50 cells. The y-axis represents the sorting index given the chosen mechanical parameters. Each coloured dot in the plot at right represents a different combination of mechanical parameters for each cell, defined fully in Reference (Revell et al., 2019). Importantly, we found a striking degree of correlation between the cell-cell affinity and sorting index, suggesting that regardless of the underlying forces, cell-cell affinity is a very good predictor of cell sorting.

Given the excellent correlation between cell-cell affinity and cell sorting regardless of the underlying forces, we made a simplifying assumption that both cell types have the same cortical tension and the same adhesion magnitude (approximated as 0.2^*γ*^_cm_ as inspired by references (Chan et al., 2017; Maître et al., 2012), where ^*γ*^_cm_ is the cortical tension), and the only parameter that was varied was interfacial tension. We further demonstrated in reference (Revell et al., 2019) that the only value that played a role in sorting was the ratio between the interfacial tensions of EPI::EPI and PrE::PrE and not the absolute values. Thus, EPI::EPI interfacial tension was a variable, and the affinity parameter, *β*, was calculated accordingly using Equation 1 in the text. The median values of pEPI and pPrE cell’s external contact angle are used to estimate the dimensionless parameter *β* given by Equation 1 in the text.

We model surface fluctuations, *ε*, as a local change in the cortical tension resulting in a protrusion from the cell surface. To achieve this, we devised a simple algorithm demonstrated in (Figure 1 of Revell et al., 2019). Each cortex element on the surface of a cell is given a randomly allocated phase *φ*, which increases linearly with time. This phase is used to determine the strength of cortex forces experienced by the cortex element, which vary sinusoidally with a period of τ/10 where *τ* is the cell cycle time. Thus, the force experienced by the element from any cortical tension interaction is modulated by a term, δ∗sin(10t/τ+ϕ). A dimensionless parameter *ε* can be formed from the ratios of *δ* in pPrE to pEPI. This oscillation in tension at a particular point in the surface causes the element to protrude from the cell surface before being pulled back in, modelling a fluctuation. The behaviour of the system can be controlled by varying the surface fluctuation amplitude. The relevant parameter is the ratio of surface fluctuations of PrE to EPI, *ε*, which we calculated from experiments as ≈ 0.35 (^*δ*^_EPI_ = *1.39* μm, ^*δ*^_PrE_ = *1.91* μm).

Throughout each simulation, the extent of sorting was probed using a numerical sorting index (Revell et al., 2019). The sorting index is the ratio of the proportion of this area occupied by PrE cells to the total external surface area of the cell aggregate (Figure 3 of Revell et al., 2019). To provide context to the values obtained from the surface sorting measure, we implemented a randomised system normalisation (Revell et al., 2019). This algorithm randomly reallocates the fates of all cells in the system after each measurement, retaining the spatial arrangement and the number of each cell type, and repeats the measurement for each arrangement 100,000 times to obtain the mean and standard deviation of the measure across all randomised systems (Figure 3 of Revell et al., 2019). The system is then reverted to the original simulation state. We can then present the results of the simulation as a sorting index defined as *SI* = $\left(X_{S}-\mu /4\sigma \right)$. Each parameterisation of the model was tested four times for all results in the paper, and the mean and standard deviation for each run is reported.

In the simulation, we set pEPI and pPrE cells size (radius) as the same based on the measurement (E3.5 pEPI/ICM: 7.22±0.69, pPrE: 7.23±1.22, E3.75 pEPI: 7.13±1.31, pPrE: 7.27±1.19, E4.0 pEPI/EPI: 6.88±0.66, pPrE/PrE: 6.26±0.83). EPI and PrE in both a half embryo and a double embryo can sort similar to a normal size embryo indicating that the total number of ICM cells, at least from 0.5 to 2 times difference, doesn’t affect EPI and PrE segregation (Saiz et al., 2016). We simulated up to 50 cells noting that the number of E3.5 ICM and E4.5 ICM is roughly 10-20 cells and 40-50 cells respectively. We approximated the proportion of both pEPI and pPrE as 50%.

Simulations were performed on the University of Cambridge Darwin HPC facility, running one simulation per core independently, using the Intel FORTRAN compiler.

##### Cell preparation and experimental setup for ICM cell/aggregate surface fluctuation analysis

mTmG^*+/-*^*Pdgfra*^*H2B-GFP/+*^ positive embryos obtained from intercrossing of *Pdgfra*^*H2B-GFP/+*^ and mTmG^*+/+*^ were used. For single ICM cell membrane dynamics study, isolated single ICM cells were transferred in the Blast drops under mineral oil on a PDL-coated glass-bottomed dish. The cells were kept for 15 minutes in a humidified incubator at 37°C and 5% CO_2_ and subsequently kept for 15 minutes in an imaging chamber at 37°C and 7% CO_2_ before imaging for purposes of equilibration. For the cytokine and inhibitor experiments, 25 ng/ml FGF2 (in-house), 1 μM PD03 or 0.01% DMSO (Thermo Fisher Scientific) was added to Blast drops. For ICM aggregates membrane dynamics study, small depressions were indented on a 60-mm dish (Thermo Fisher Scientific) lid by a sterilised aggregation needle (BLS Ltd) and covered with Blast drops (one depression per one drop) overlaying with mineral oil. Three isolated E3.75 ICMs were disposed to make a triangle in a small depression and kept for one hour in a humidified incubator at 37°C and 5% CO_2_. Aggregated ICMs were transferred in the Blast drops under mineral oil on a PDL-coated glass-bottomed dish. Before transferring ICM cells or aggregated ICMs to Blast drops, the drops under mineral oil were buffered in a humid incubator at 37°C and 5% CO_2_ for at least 30 minutes. Live images were acquired using Leica TCS Sp5 Confocal microscopy on the single middle z-slice through the cell every ten seconds for ten minutes with three channels (488 nm excitation for *Pdgfra*^*H2B-GFP/+*^ reporter, 567 nm excitation for membrane [mTmG] and bright field). For ICM aggregates membrane dynamics study, live images were taken on the several z-slice through the aggregates every 20 seconds for ten minutes.

##### Cell lineage classification

The mean intensity of *Pdgfra*^H2B-GFP^ was measured by Fiji. ICM cell lineages were determined by their GFP signal. The E3.5 cells with GFP positive were classified as pPrE. The E3.5 cells with GFP negative were classified as ICM/pPrE cells. The E3.75 cells with the top 40% of GFP intensity were classified as pPrE, the E3.75 cells with the bottom 40% of GFP intensity were classified as pEPI. The E4.0 cells with GFP positive were classified as PrE. The E4.0 cells with GFP negative were classified as EPI. Mitotic cells judging by H2B-GFP morphologies were removed from the analysis.

##### Quantification of surface fluctuations

Each cell’s live imaging data was cropped and registered with StackReg plugin (Thévenaz et al., 1998) using Fiji. The centroid of the first images was used as the centre of the new coordinates system and linear interpolation (See Figures 3B and S3B). The position of the cell membrane (tdTomato signal of mTmG) was identified and converted from Cartesian coordinates into polar coordinates. The boundary coordinate plots of the radius versus the angular coordinate (*θ*) were detrended to set the average radial value is zero. This normalises for differences in the cell size and controls for small fluctuations of the focal plane in the *z*-axis. The variation in time (*V*_T_) was calculated using Equation 4, *ρ* : distance from the centre of a cell.

(Equation 4) $$V_{\text{T}}=\bar{{\left(SD\left(\left\{\rho_{\text{j}\;}\left(t_{1}\right),\rho_{\text{j}\;}\left(t_{2}\right),\ldots ,\rho_{\text{j}\;}\left(t_{\text{N}}\right)\right\}\right)\right)}_{\text{j}=1:\text{M}}}$$

#### Surface fluctuation analysis of ICM aggregates

Surface fluctuations on outside cells were scored from 1 to 5, with 1 being no observable fluctuations and 5 being significant observable fluctuations, using only mTmG time-lapse images so as not to identify each cell lineages. A high score indicates that the cell has a dynamic cell membrane movement. This analysis was done single-blind by AY and KC.

#### Surface fluctuation analysis of ES cells

For CA-EZR-IRES-mCherry ES cells were plated for 24 hours in N2B27+2i+LIF at 2 × 10^4^ cells/cm^2^, and the media were changed to N2B27+2i+LIF with or without 1 μg Dox. Then, cells were dissociated into a single-cell suspension using Accutase and suspended in 100 μl each culture media with 0.01% CellMask™ Green Plasma membrane Stain. The media were buffered in the incubation chamber for at least 30 minutes before suspension. The analysis was performed in the same manner as ICM cell surface fluctuation described above.

#### Cell shape analysis of pEPI and pPrE in a ICM aggregates and ES cells aggregates

For calculation of shape index in the ICM, the analyst was blinded to the fluorescent channel identifying cell type. The perimeter and area of each cell was found using Fiji. The shape index was calculated as the perimeter divided by the square root of the area of the cell. All shape analyses were performed using z-stacks and the slice with the maximum area of the cell was used for the analysis. Coefficient of variation was calculated as the standard deviation of the shape index divided by the mean of the shape index of 15 frames over time (30 seconds between frames).

#### Vertex models: Exploring the connection between shape-induced tension fluctuations and de-mixing, in a confluent bulk of cells

Recently, cell shape index in a vertex model has emerged as a faithful indicator of its fluidity (Bi et al., 2015, 2016; Farhadifar et al., 2007; Merkel and Manning, 2018; Staple et al., 2010). In recent work by some of us, cells with difference shapes (and thereby fluidity) as high as in our experiments could produce robust small-scale patterning and demixing (Sahu et al., 2020). The origin of the small-scale demixing was attributed to differential neighbour exchange barriers at the heterotypic interface. Even though such barriers were not yet quantified systematically in the bulk of 3D tissues, the response to local perturbations along high-tension interfaces was found to be very similar for both dimensions (Sahu et al., 2021; Sussman et al., 2018). Hence, we studied the interface formation in a mixture composed of two different cell shapes and with no explicit tension, to probe if a similar small-scale demixing as reported in Sahu et al. (2020) existed in 3D.

Here, we used the same formula of demixing parameter, in which a value of zero and unity correspond to the minimum and maximum segregation in the tissue. We simulated a mixture composed of 216 polyhedral cells with equally sized sub-components using a 3D self-propelled Voronoi model with periodic boundary conditions. The preferred cell shape index $s_{0}$ was a non-dimensionalised version of the preferred surface area $S_{0}$ and preferred volume $S_{0}=S_{0}/V_{0}^{2/3}$ $V_{0}$ i.e. A tissue could transition from being solid-like to fluid-like by increasing the preferred shape index to a value higher than ∼5.41 in 3D (Merkel and Manning, 2018). Considering the average tissue fluidity to be a free parameter, we studied for increasingly fluid-like average shape values of 5.60, 5.75 and 5.90. We fixed the shape disparity to the experimentally obtained value. As shape quantification was easier for 2D cross-sectional images, we used the 2D values to infer the 3D estimate of shape difference as ∼0.8 using the work by Sharp et al. (2019). For such high differences in shape values, the volume incompressibility $\left(K_{V}\right)$ needs to be high in order to prevent the size disparity from affecting the patterning. Therefore, we set the $K_{V}$ to 10 while $V_{0}=1$. From the 2D work, one should expect an increase in incompressibility to have a negligible effect on the cellular dynamics.

The natural timescale for our systems was given by $\tilde{t}=1/\left(K_{V}.V_{0}^{4/3}\right)$ which was less than unity for our choice of parameters. Therefore the integration time stepsize was chosen as $\Delta t=0.01\tilde{t}$. For evolving the cellular positions, we used the self-propulsion dynamics where in, the magnitude of self-propulsion speed $\left(v_{0}\right)$ is set to 0.1 and rotational diffusion coefficient $\left(D_{r}\right)$ to 1.0. For such high values of rotational diffusivity the transition to brownian regime happened rather quickly i.e $t/\tilde{t}>1/D_{r}$. The typical self-diffusivity timescale in our model for a fluid-like shape index of 5.5 was found to be $1\times 10^{4}\tilde{t}$. We run for a total of $6\times 10^{4}\tilde{t}$ that is long enough for an average fluid-like cell to explore the box sidelength of 6 cells.

The initial and final snapshots of a randomly mixed initialization are shown in Figure 7C. While the mixed initial frame has negligible de-mixing, the final snapshots do have a small-scale patterning. We then quantified the demixing for mixtures with increasing average fluidity, averaged over 250 different initializations each and observe the existence of a small but robust value of segregation that seems to have systemic increase with tissue fluidity. This confirmed that as in the 2D set up, 3D mixtures can also undergo small-scale demixing due to differences in their cell shapes.

To probe for a relationship between the shape index and surface tension fluctuations, we used an energy-minimized configuration of a solid and fluid mixture from Sahu et al. (2020) that was produced by using the open source CellGPU code (Sussman, 2017). Looking closely at the fluid subtype, we found that it has a significant number of edges with negative tension (highlighted in blue) as opposed to the solid subtype where such edges were negligible in number (Figure 7D). This might be the crucial link between the more fluid-like subtype having more *extensile* edges and hence in a non-confluent environment manifests as a greater number of blebs.

#### CA-EZR overexpression and imaging

For CA-EZR overexpressing experiments, cells were plated for 24 hours in 2i+LIF at 2 × 10^4^ cells/cm^2^, and the media were changed to 2i+LIF in the presence of 1 μg Dox. For the imaging of pERM in single CA-EZR cells, cells were cultured in 2i+LIF in the presence of 1 μg Dox for 24 hours prior to being plated onto imaging dishes (μ-Dish 35 mm ibidi dish [81156]). After six hours, the cells were fixed using 4% PFA in CSB for 15 minutes and then permeabilised in with 0.1% Triton in CSB. Blocking was then performed using 2% FBS, 2% BSA in PBS with 0.1% Triton-X for 45 minutes. Cells were then incubated for 90 minutes with the primary antibody in the same buffer as used for blocking. Cells were then washed three times for five minutes with PBS containing 0.1% Triton-X. Secondary antibodies were added together with Alexa Fluor™ 647 Phalloidin for one hour in the same buffer as used for blocking and primary antibody incubation. Cells were washed with PBS containing 0.1% Triton-X three times for five minutes before a final wash in PBS.

#### ES cells aggregation

ES cells harvested on tissue culture plates were detached using Accutase. The cells were suspended in FBS (GE Healthcare Life Sciences) containing suspension media, and cell concentration was determined. Suspended cells were centrifuged at 1400 rpm for three minutes, pelleted and re-suspended in 2i+LIF in the absence or presence of 1 μg/ml Dox. H2B-BFP and CA-EZR-IRES-mCherry ES cell lines were mixed well in a universal tube (Scientific Laboratory Supplies Ltd) at 1:1 ratio and then plated 200 μl per well in round-bottomed low-adhesion 96-well plates (CELLSTAR) with 300 cells per well. After 30 hours culture, aggregated ES cells were collected by mouth pipette, briefly rinsed in PBS, fixed with 4% PFA for 20 minutes at room temperature, rinsed in PBS/PVP, and then incubated briefly in increasing concentrations of Vectashield before mounting on glass slides (Thermo Fisher Scientific) in small drops of concentrated Vectashield. Subsequently, coverslips with Vaseline (Unilever) spacer were mounted and sealed with nail varnish. FBS containing suspension media was composed of Glasgow’s minimum essential medium (GMEM) with 10% batch-tested FBS, 1× MEM non-essential amino acids (NEAA; Sigma), 1 mM sodium pyruvate (Sigma), and 1 mM L-glutamine, 0.1 mM 2-mercaptoethanol. For calculation of the average radial distance from the centre, *R*, we assessed the distribution of mCherry levels in the cells. The bottom half of the distribution was assumed to be the control cells and was discarded. In the top 50% expressing cells, the distributions were split into a bottom 1/3, a middle 1/3, and a top 1/3. These were considered, respectively, as the low-, mid-, and high-expressing cells. These were then binarized and used in the formula shown in Figure 4F to find *R*.

Rex1-GFPd2/Gap43-mCherry ES cells and R26-Confetti ES cells were used to form aggregates containing siERM ES cells or siACTN4 ES cells and negative control siRNA (NC) cells. siRNA was transfected 12-16 hours before aggregate formation. Aggregation was performed as above with a total seeding density of 40 cells or 80 cells in a 1:1 (siERM/siACTN4: siNC) ratio in N2B27+2i+LIF. For live imaging, aggregates were collected by mouth pipette after 7-8 hours and seeded on N2B27+2i+LIF on PDL-coated dishes.

#### RNA interference

1 x 10^4^ cells tdTomato ES cells were seeded on 0.1% gelatine coated 24-well plates. Cells were transfected with 15 μM of either the targeting (5 μM each SMARTpool siGENOME Mouse Ezrin [M-046568-01-0005], Radixin [M-047230-01-0005], Moesin [M-044428-01-0005]; Dharmacon), actinin alpha4 (M-049970-00-0005, Dharmacon) or control siRNA (D-001210-02-05, Dharmacon) with Lipofectamine RNAiMAX Transfection Reagent (Thermo Fisher Scientific) for overnight. The media were changed the next day. The transfected cells were utilised for cell shape imaging or chimaera assay. Knockdown efficiency was checked using RT-qPCR.

#### RNA extraction and cDNA synthesis

Total RNA was prepared with the RNeasy Kit (Qiagen), and reverse transcribed using SuperScriptII (Invitrogen) according to the manufacturer's protocol. Real-time PCR was performed using TaqMan Fast Universal Master Mix and TaqMan Gene Expression assays (Applied Biosystems). *Gapdh* was used as an endogenous control (Applied Biosystems). For siRNA experiments, the data were further normalised to control cell line. The TaqMan Gene Expression assays IDs were Mm00447761_m1; *Ezrin*, Mm00447889_m1; *Moesin*, Mm01177363_m1; *Radixin*, Mm00502489_m1*; α-actinin4*.

#### Generation of chimaeras

ES cells (three-five cells per embryo) were injected into 8 cell morulae (E2.5) or early blastocysts (E3.5) via a laser-generated perforation in the zona pellucida using XYClone (Hamilton Thorne Biosciences). Injected embryos were cultured in Blast or N2B27 for 1.5 or 0.5 days, the reach the equivalent of E4.0 blastocysts at 37°C and 5% CO_2_.

#### Membrane tension measurements by optical trap

E3.75 *Pdgfra*^*H2B-GFP/+*^ positive embryos obtained from intercrossing of *Pdgfra*^*H2B-GFP/+*^ and CD-1 were used. Isolated single ICM cells were transferred in the M2 drops on a PDL-coated μ-Dish 35 mm ibidi dish. The dishes were exposed to plasma surface treatment (Pico, diener). 50 μg/ml PDL drop was put at the centre of the dish. The coated dishes were incubated for 30 minutes ∼ one hour at room temperature. The PDL drop was washed with M2 medium for three times. The cells were kept for 15 minutes in a humidified incubator at 37°C and 5% CO_2_. M2 medium concanavalin-A coated (50 μg/ml) carboxyl latex beads (1.9 μm diameter, Thermo Fisher Scientific [C37278]) were added to the drop prior to measurement.

A tether pulling assay was then performed using a homemade built optical tweezer (4W1064nm Laser Quantum Ventus) on an inverted microscope (Nikon Eclipse TE2000-U) equipped with a motorised stage (PRIOR Proscan). During the measurements, the position of the bead was recorded at a 90 milliseconds interval rate in the bright field using a 100x oil immersion objective (CFI Plan Fluor DLL, Nikon). pEPI and pPrE cells were identified by their GFP signals. The trap force was later calculated based on a product of the bead displacement (estimated using a custom-made ImageJ plugin) and of the trap stiffness (which was extracted using the method described in Lieber et al., 2013).

For the measurement of the variance of the trap force overtime, ∼10^6^ ES cells were plated 16 hours prior to the measurement onto μ-Dish 35 mm ibidi dishes in N2B27. Membrane tension was then measured following the method described above. Of note, here membrane tension was continuously measured on the same cell using a single tether for approximatively five minutes. The number of blebs was then manually counted during the analysis, only blebs forming or retracting during the measurements were taken into account. Data in which the formation of blebs was physically interfering with the tether were excluded from the analysis.

##### Direct stochastic optical reconstruction microscopy (dSTORM) imaging and analysis sample preparation

E3.75 *Pdgfra*^*H2B-GFP/+*^ positive embryos obtained from intercrossing of *Pdgfra*^*H2B-GFP/+*^ and CD-1 were used. Isolated pPrE and pEPI cells were cultured in a PDL-coated small drop of N2B27 with or without 25 ng/ml FGF2 on 35 mm glass-bottom dishes (MatTek; P35G-0.170-14-C) at 37°C and 5% CO_2_ for 45 minutes. The samples were fixed and stained in the same manner as pERM staining described in the STAR Methods. Primary and secondary antibodies were diluted in PBST (PBS supplemented with 0.1% Tween). For the secondary antibody incubation, cells were first incubated with AlexaFluor donkey anti-goat 488 (Thermo Fisher, 1:1000) followed by incubation with Hoechst 33342 and AlexaFluor647 Phalloidin (Thermo Fisher, 1:200) diluted in PBST for another hour. Cells were then gently washed three times with PBST. The imaging dishes were then filled with STORM buffer (50 mM Tris pH 7.5, 10 mM NaCl, 10% glucose (w/v), 27 mM MEA, 40 μg/mL catalase, 5 U/mL pyranose oxidase and 2 mM cyclooctatetraene) and imaged immediately.

#### Imaging and analysis

dSTORM imaging was performed on a commercial Zeiss Elyra 7 microscope. F-actin was imaged in the cellular mid-plane through a 63 x 1.46 NA alpha Plan-Apochromat oil-immersion objective lens and a 1x tube lens under 642 nm (100% laser power) and 405 nm (0-2% laser power) excitation. For each super-resolution image of F-actin, a Hoechst 33342 (405 nm excitation) and GFP (488 nm excitation) snapshot was also acquired. Fluorescence was captured on a sCMOS camera using 20 milliseconds integration time. A total of 15000 frames were typically acquired for super resolution image reconstruction. Reconstructions were generated using the in-built ZEN Black software (Zeiss). Briefly, single molecule localisation was estimated using a multiple object 2D Gaussian fitting routine, accounting for overlapping fluorophores. Data was post-processed to correct for mechanical drift during the acquisition by applying a model-based cross-correlation method and localisations with uncertainties >50 nm were then removed from the data-sets. Cortex thickness measurements were performed on the super-resolution images using a custom-written MATLAB script (described in Serres et al., 2020). Briefly, the cell cortex for each super-resolution data set was manually estimated using a custom MATLAB GUI. The cell cortex was then automatically detected by calculating the maximum intensity peak of the transverse intensity profile along the cell periphery at each pixel along with the user-supplied cortex co-ordinates. The cortex was then straightened using cubic spline fitting, and line scans of the intensity profiles across well-defined cortical regions were performed, from which full-width at half-maximum (FWHM) values were used to estimate cortical thickness.

### Quantification and statistical analysis

#### Statistical analysis

For all statistical analysis in the paper, unless otherwise indicated in corresponding figure legends, n-way ANOVA was used to calculate P-values to establish significant changes between any two means. Details of each ANOVA stated in figure legends: for example, variables used for the n-way ANOVA could be ‘replicate number’, ‘cell type’ (e.g. EPI and PrE) and when relevant ‘small molecule treatment’. There was frequently a significant interaction effect for ‘experiment number’ in the embryo experiments because of experimental variability due to uncertainty in mating times intrinsic to embryo work, so the p-value reporting the significance of this effect is not reported throughout the paper. When there was no significant interaction effect for experimental variability and no ‘treatment’, the p-value reflects a one-way ANOVA. The p-value reported is for ‘cell type’ or ‘small molecule treatment’ depending on the experiment, as indicated in the figure caption. The midline is mean of overall experiments, and error bars represent standard deviation over all experiments.

## Data Availability

The scRNA-seq datasets generated during this study are available at NCBI’s Gene Expression Omnibus (GEO) Accession Number GSE148462. Codes and any other data that support the findings of the study are available from the corresponding authors upon request.

## Supplemental information

Table S1. Highly modulated actin-cytoskeleton-related genes in ICM cells through E3.5 to E4.5, related to Figure 1 (A) Actin-cytoskeleton genes list based on Go terms. (B) Actin-cytoskeleton genes based on the listed Go terms. (C) Highly modulated actin-cytoskeleton-related genes in ICM cells through E3.5 to E4.5.

Table S2. NC-, ERM-, or ACTN4-siRNA-treated ESC location and numbers in the chimaera blastocyst, related to Figure 6
