# Cell surface fluctuations regulate early embryonic lineage sorting

**DOI:** 10.1016/j.cell.2022.03.015

**Published:** 2022-03-31

**Authors:** Ayaka Yanagida, Elena Corujo-Simon, Christopher K. Revell, Preeti Sahu, Giuliano G. Stirparo, Irene M. Aspalter, Alex K. Winkel, Ruby Peters, Henry De Belly, Davide A.D. Cassani, Sarra Achouri, Raphael Blumenfeld, Kristian Franze, Edouard Hannezo, Ewa K. Paluch, Jennifer Nichols, Kevin J. Chalut

(Cell *185*, 777–793.e1–e20; March 3, 2022)

In the original article, the authors cited a Kim et al., 2021, study when discussing recent work on tissue fluidization. They intended for this citation to reference work from Campàs and colleagues but inadvertently included a different Kim et al., 2021, study from Style and colleagues in the references instead. This error has now been corrected in the online version of the paper. We apologize for any confusion this may have caused.

